# Synthetic microbial community improves chicken intestinal homeostasis and provokes anti-*Salmonella* immunity mediated by segmented filamentous bacteria

**DOI:** 10.1093/ismejo/wraf076

**Published:** 2025-04-23

**Authors:** Meihong Zhang, Suxin Shi, Yimei Feng, Fengwenhui Zhang, Yuxuan Xiao, Xin Li, Xingliang Pan, Yuqing Feng, Dan Liu, Yuming Guo, Yongfei Hu

**Affiliations:** State Key Laboratory of Animal Nutrition and Feeding, College of Animal Science and Technology, China Agricultural University, Haidian District, Beijing 100193, China; State Key Laboratory of Animal Nutrition and Feeding, College of Animal Science and Technology, China Agricultural University, Haidian District, Beijing 100193, China; State Key Laboratory of Animal Nutrition and Feeding, College of Animal Science and Technology, China Agricultural University, Haidian District, Beijing 100193, China; State Key Laboratory of Animal Nutrition and Feeding, College of Animal Science and Technology, China Agricultural University, Haidian District, Beijing 100193, China; State Key Laboratory of Animal Nutrition and Feeding, College of Animal Science and Technology, China Agricultural University, Haidian District, Beijing 100193, China; State Key Laboratory of Animal Nutrition and Feeding, College of Animal Science and Technology, China Agricultural University, Haidian District, Beijing 100193, China; Department of Microbiology, Beijing General Station of Animal Husbandry, Chaoyang District, Beijing 100107, China; State Key Laboratory of Animal Nutrition and Feeding, College of Animal Science and Technology, China Agricultural University, Haidian District, Beijing 100193, China; State Key Laboratory of Animal Nutrition and Feeding, College of Animal Science and Technology, China Agricultural University, Haidian District, Beijing 100193, China; State Key Laboratory of Animal Nutrition and Feeding, College of Animal Science and Technology, China Agricultural University, Haidian District, Beijing 100193, China; State Key Laboratory of Animal Nutrition and Feeding, College of Animal Science and Technology, China Agricultural University, Haidian District, Beijing 100193, China

**Keywords:** synthetic microbial community, intestinal homeostasis, segmented filamentous bacteria, *Salmonella*

## Abstract

Applying synthetic microbial communities to manipulate the gut microbiota is a promising manner for reshaping the chicken gut microbial community. However, it remains elusive the role of a designed microbial community in chicken physiological metabolism and immune responses. In this study, we constructed a 10-member synthetic microbial community (SynCom^Bac10^) that recapitulated the phylogenetic diversity and functional capability of adult chicken intestinal microbiota. We found that early-life SynCom^Bac10^ exposure significantly enhanced chicken growth performance and facilitated the maturation of both the intestinal epithelial barrier function and the gut microbiota. Additionally, SynCom^Bac10^ promoted the pre-colonization and growth of segmented filamentous bacteria (SFB), which in turn induced Th17 cell-mediated immune responses, thereby conferring resistance to *Salmonella* infection. Through metagenomic sequencing, we assembled the genomes of two distinct species of SFB from the chicken gut microbiota, which displayed common metabolic deficiencies with SFB of other host origins. In silico analyses indicated that the SynCom^Bac10^-stimulated early establishment of SFB in the chicken intestine was likely through SynCom^Bac10^-derived metabolite cross-feeding. Our study demonstrated the pivotal role of a designed microbial consortium in promoting chicken gut homeostasis and anti-infection immunity, providing a new avenue for engineering chicken gut microbiota.

## Graphical Abstract

**Graphical Abstract:**
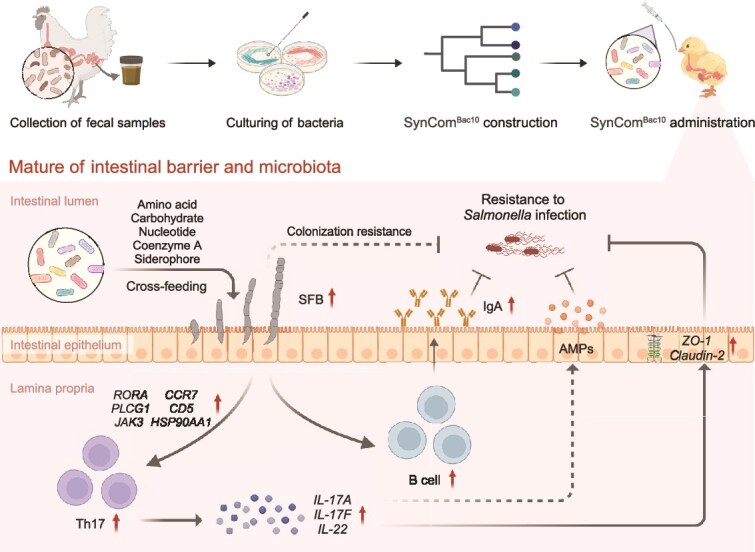

## Introduction

The chicken gastrointestinal tract hosts a complex microbial community that plays a critical role in maintaining chicken intestinal homeostasis, thereby facilitating better nutrient digestion and absorption, and strengthening intestinal barrier and immune function [1]. Compared with chickens raised under conventional conditions, germ-free or low-bacterial-load chickens exhibit reduced villus height, gut weight, length, and wall thickness, and compromised immune function, which increases susceptibility of the host to enteric and non-enteric infections [2–4]. Gut commensals with immunomodulatory role in chickens, as well as in other animals, therefore earn special attention. For instance, segmented filamentous bacteria (SFB) have been shown to trigger differentiation of Th17 cells, which play crucial roles in pathogen defense [5], and *Bacteroides fragilis* and *Clostridium* clusters XIVa and IV are demonstrated to stimulate development of Treg cells, which are essential for maintaining immune tolerance and preventing excessive immune responses [6, 7].

Although the exact origin of the avian gut microbiota remains unclear, maternal microorganisms are considered a significant source for the initial microbial colonization of the gastrointestinal tract in birds. The chicken early microbial community appears less resistant and stable, and once disrupted by external stimuli, it may cause irreversible damage to later chicken growth and production [8]. Along with the chicken’s growth, their gut microbiota evolves dynamically, transitioning from an initially simple community dominated by facultative anaerobes to a more stable and complex community that includes obligate anaerobes. Studies have divided the development of the chicken gut microbiota into different succession patterns [9, 10], with a consistent notion that the first week post-hatching is the optimal window for microbiota intervention [10–12], as this stage is pivotal for the establishment of early colonizers and the resulting maturation of the immune system. Nevertheless, in modern poultry farming practices, the prevention of direct contact between the mother and chicks disrupts the early transmission of mature microbes to the offspring. Thus, it is attractive to use functional microbes from the adults as a bird early life inoculum to reconstruct the avian gut microbiota.

Currently, the most direct manner to manipulate or reconstruct the animal/human gut microbiota is through fecal microbiota transplantation (FMT). In chickens, FMT has been shown to improve growth performance, intestinal barrier, and immune system function [13, 14]. Despite the promising outcomes, large-scale application of complex and undefined stool material for maintaining or enhancing animal or human health remains practically challenging [15]. For example, using such a heterogeneous mixture poses risks of transferring pathogenic or undesirable microbes to recipients. In addition, the composition of the transplanted microbiota can vary significantly between donors, compromising functional stability. Moreover, FMT requires the collection and processing of fecal material from donors, which is less practical for large-scale use. In contrast, a synthetic microbial community with defined culturable microbes ensures enhanced safety, controllability, and reproducibility [16]. Different synthetic microbial communities were reported to enhance the host to resist pathogens, including *Salmonella*, *Clostridium difficile*, *Listeria monocytogenes*, and *Enterobacteriaceae* [17–20]. Besides, synthetic communities were used for treating chronic immune-mediated colitis and *Gardnerella vaginalis*-induced bacterial vaginosis in mice and humans [21, 22]. Recently, in chickens, a nine-member synthetic microbial community representing the most prevalent phyla from chicken intestine was demonstrated to modulate host immune responses [23]. These studies fully support that defined synthetic microbial communities can partially exert the function of the complex gut microbiota, especially in immunomodulation, and synthetic consortium represents a promising strategy to engineer the host gut microbiota.

**Figure 1:**
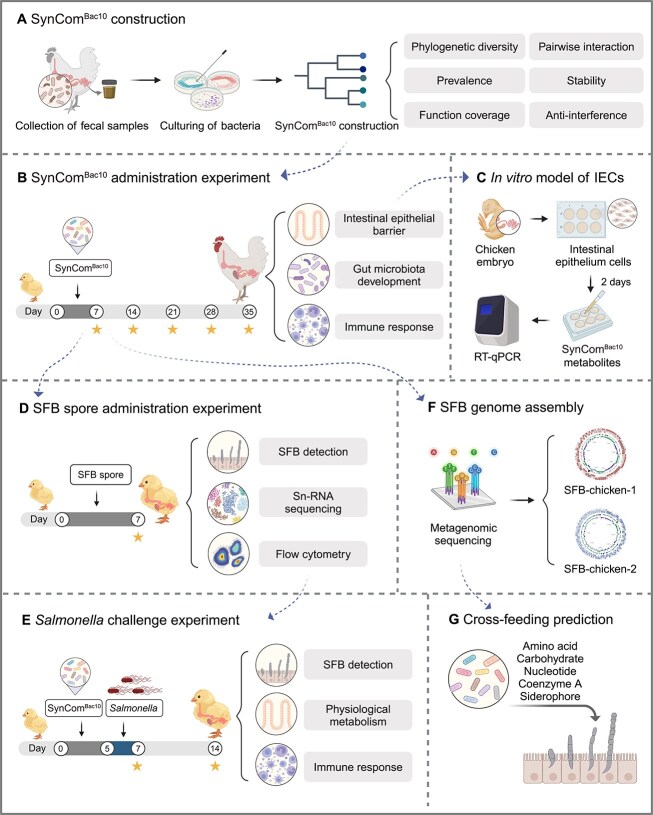
Overview of the whole experimental design. Created with BioRender.com.

In this study, to investigate the role of a synthetic microbial community as an early life gut inoculum in the development of chicken gut microbiota and its effects on physiology and metabolism, and immune system function, we constructed a ten-member synthetic microbial community (referred to as “SynCom^Bac10^”), which mimics the structure and function of the adult chicken gut microbiome (Fig. 1). We demonstrated that SynCom^Bac10^ played a positive role in improving chicken intestinal homeostasis and promoted the early establishment of SFB in chicken gut. We further demonstrated that SynCom^Bac10^-stimulated SFB assisted chickens in resisting *Salmonella* infection via Th17 cell-mediated immune responses. Additionally, we assembled the genomes of two distinct SFB species from the SynCom^Bac10^-chicken gut contents and proposed that the SynCom^Bac10^ promoted SFB growth through crucial metabolite cross-feeding. Our findings provide compelling evidence that early life inoculation with SynCom^Bac10^ is a promising strategy to enhance gut homeostasis and overall health in chickens.

## Materials and methods

### Ethics approval

All animal experiments were approved by the Institutional Animal Care and Use Committee of the China Agricultural University (Approval No. AW62214207–1-01).

### Culturomics

A total of 50 healthy 35-day-old chickens, showing no visible signs of disease or stress and raised under standard farming conditions at Zhuozhou farm (Hebei, China), were selected for sampling of their gut contents. The samples were immediately transported to CAST MicroLab (China Agricultural University, Beijing, China) using dry ice for culturing bacteria. More details for culturomics are provided in Supplementary Materials.

### Preparation of SynCom^Bac10^ members for *in vitro* experiments

The 10 SynCom^Bac10^ members were prepared from frozen culture stocks. To ensure the same proportion of each strain in SynCom^Bac10^, a simplified approach to community reconstitution was proposed. Each strain grown in the liquid culture medium was diluted 1:10 into fresh growth medium every 24 hours for 3 days. Following a 72-hour growth period at 37°C without shaking under anaerobic conditions, the optical density of each strain was assessed at 600 nm (OD_600_), and was then diluted to a final OD_600_ of 0.1 using fresh growth medium. Afterward, they were centrifuged at 6000 × g for 5 minutes, washed three times, and resuspended with an equal volume of PBS for further experiments. Details for bacterial growth measurement, pairwise co-culture assay, community growth, and pathogen interference assays are provided in Supplementary Materials.

### Preparation of SynCom^Bac10^ for in vivo experiments

The SynCom^Bac10^ members were cultured as described above. Based on OD_600_ measurements and colony-forming units (CFUs) counting, studies have demonstrated that an OD_600_ of 1.3 corresponds to a cell concentration of 10^9^ cells per mL for *Escherichia coli* [24]. According to this estimate, each bacterial culture was concentrated and adjusted to a final OD_600_ of 1.3 after a 72-hour growth period. Afterward, they were mixed together, centrifuged, washed, and resuspended in a 10-fold volume of PBS containing 20% glycerol. An aliquot of 200 μl suspension was orally administered in the next in vivo experiment, ensuring that each chicken received 10^8^ bacterial cells each day. More details for SynCom^Bac10^ administration, *Salmonella* challenge, SFB spore preparation, and administration are provided in Supplementary Materials.

### Isolation of primary chicken embryo intestinal epithelium cells and SynCom^Bac10^ treatment

Primary chicken embryo intestinal epithelium cells (IECs) were isolated from 17-day-old pathogen-free chicken embryonated eggs and were then treated with the SynCom^Bac10^ culture supernatant. More details for isolation of primary chicken embryo IECs and SynCom^Bac10^ treatment are provided in Supplementary Materials.

### Whole-genome sequencing

All SynCom^Bac10^ members were sequenced using the HiSeq System (Illumina) served by the Institute of Microbiology of the Chinese Academy of Sciences (Beijing, China). Details for bioinformatics analysis are provided in Supplementary Materials.

### DNA extraction and absolute real-time quantitative PCR

Bacterial DNA was extracted using Bacteria Genomic DNA Kit (CWBIO, Beijing, China). DNA concentration was subsequently determined by EPOCH2 Microplate Readers (BioTek). A targeted DNA concentration was prepared by PCR amplification using DNA isolated from targeted microbial strain. All specific primers were synthesized and listed in Table S1. More details for absolute real-time quantitative PCR (RT-qPCR) are provided in Supplementary Materials.

### 16S rRNA gene amplicon sequencing and microbiome analysis

The QIAamp Fast DNA Stool Mini Kit (Qiagen, Germany) was used to extract the bacterial genomic DNA from the chicken ileal or cecal contents. Qualified DNA samples were sequenced targeting the V3-V4 variable region of the 16S rRNA genes. The paired-end sequencing was performed using NovaSeq System (Illumina) served by Majorbio Bio-pharm Technology Co., Ltd. (Shanghai, China). Details for bioinformatics analysis are provided in Supplementary Materials.

### Transcriptome sequencing analysis

Total RNA was extracted from ileal tissue utilizing TRIzol reagent. The integrity of RNA was assessed using an Agilent 5300 Bioanalyzer, and purity and concentration were quantified with a Nanodrop 2000 spectrophotometer (Thermo Scientific, USA). Construction and sequencing of the cDNA libraries were performed using NovaSeq System (Illumina) at Majorbio Bio-pharm Technology Co., Ltd. (Shanghai, China). Details for bioinformatics analysis are provided in Supplementary Materials.

### Metabolite measurement and analysis

Metabolites from serum or microbial supernatants were measured using untargeted metabolomics with a liquid chromatography-mass spectrometry system at Metware Biotechnology Co., Ltd (Wuhan, China). Details for bioinformatics analysis are provided in Supplementary Materials.

### Single-nucleus RNA sequencing

Frozen ileal mucosa was homogenized, filtered, and centrifuged to isolate nuclei for library preparation. The 10 × genomics system was employed for library preparation, and libraries were sequenced on NovaSeq system (Illumina) by Kidio Biotechnology Co., Ltd. (Guangzhou, China). Details for bioinformatics analysis are provided in Supplementary Materials.

### Preparation of lymphocytes and flow cytometry

The preparation of single-cell suspensions from the terminal ileum and the intracellular staining of *IL-17A* were performed as described in previous studies [25, 26]. The antibodies used in the flow cytometry assays are provided in Supplementary Materials. Cells were acquired with a spectral cell analyzer (SONY ID7000), and analysis was performed using FlowJo software.

### RNA extraction and real-time quantitative PCR

Total RNA of ileal tissues or IECs was extracted using TRIzol method, and RNA reverse transcription and RT-qPCR were performed with TaKaRa reagents. The RT-qPCR was conducted in a QuantStudio 7 Flex Real-Time PCR System (Applied Biosystems, USA). Relative expression levels of each mRNA were analyzed using the 2^−ΔΔCt^ method with normalization to *β-actin* as the reference standard. The primers used were synthesized and listed in Table S2.

### Microscopic observation

The terminal ileum samples were processed for hematoxylin and eosin (HE) staining, Gram staining, and scanning electron microscopy (SEM) to characterize SFB morphology. More details for microscopic observation are provided in Supplementary Materials.

### Serum immunity and biochemical parameters

The immunoglobulin A (IgA) and immunoglobulin G (IgG) levels of serum were determined using commercial ELISA kits (TIANGEN Biotech Co. Ltd., Beijing, China). The detection of aspartate aminotransferase (AST) and alanine aminotransferase (ALT) in serum was performed using a fully automatic biochemical analyzer (BK-200VET).

### Metagenomic sequencing and chicken SFB genome assembly

The metagenomic DNA from the terminal ileum contents of 7-day-old SynCom^Bac10^ chickens was extracted using a QIAGEN DNeasy PowerSoil Pro Kit (Qiagen Ltd., Dusseldorf, North Rhine-Westphalia, Germany) and sequenced using the DNBSEQ-T7 System (Ling En Biotechnology Co., Ltd., Shanghai, China). A total of 102 995 204 shotgun metagenomic sequencing read pairs were obtained. Following assembly, 124 673 contigs were generated and used for genome binning. More details for bioinformatics analysis, SFB genome assembly, and genomic analysis are provided in Supplementary Materials.

### Statistical analysis

Statistical analysis was performed using IBM SPSS Statistics (SPSS Inc., Chicago, IL, USA) and all data were shown as means ± standard error. For comparisons between two groups, we employed either the Student's *t*-test or the Wilcoxon rank test to assess statistical significance; for data involving more than two groups, we used one-way analysis of variance (ANOVA) or non-parametric Kruskal–Wallis test. A *P* value < .05 was established as the threshold for statistical significance, and *P* values ranging from .05 to .10 were interpreted as indicating a trend.

## Results

### Construction of a synthetic microbial community with a similar structure and function to adult chicken gut microbiome

**Figure 2:**
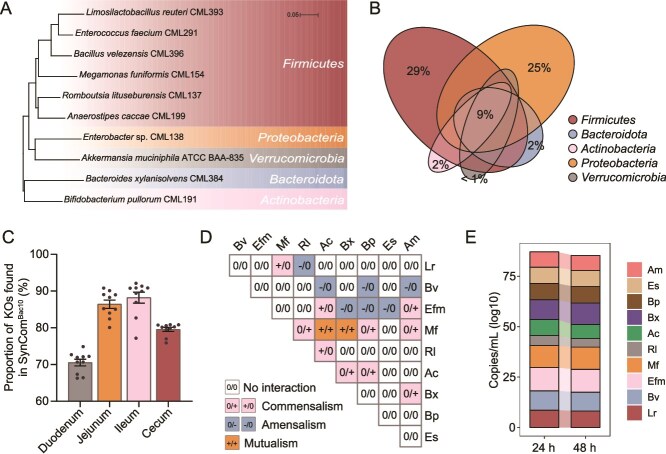
Growth analysis of SynCom^Bac10^ strains *in vitro* experiments. (A) Phylogenetic relationship of the SynCom^Bac10^ members based on the 16S rRNA genes. Scale bar: 0.05 indicates that each unit length on the phylogenetic tree corresponds to 0.05 nucleotide substitutions. (B) Euler diagram illustrating a number of KOs and overlap of the entire consortium as grouped by bacterial phyla. Size of overlap between ellipses represents a number of KOs that are shared, with comparisons between individual strains indicated as a percentage of the total number of KOs. (C) The proportions of KOs represented by the SynCom^Bac10^ in the microbial community of the duodenum, jejunum, ileum, and cecum of 35-day-old donor chickens. (D) Changes in the absolute abundance of a strain in co-culture relative to monoculture were quantified, leading to the creation of a pairwise interaction matrix. (E) SynCom^Bac10^ composition was analyzed after 24 and 48 hours by comparing the absolute abundance of 10 strains in GAM medium using RT-qPCR. The strain name abbreviations are as follows: *L. reuteri* CML393, Lr; *Bacillus velezensis* CML396, Bv; *E. faecium* CML291, Efm; *Megamonas funiformis* CML154, mf; *Romboutsia lituseburensis* CML137, Rl; *Anaerostipes caccae* CML199, Ac; *Bacteroides xylanisolvens* CML384, Bx; *B. pullorum* CML191, Bp; *Enterobacter* sp. CML138, Es; *Akkermansia muciniphila* ATCC BAA-835, Am.

To establish a synthetic microbial community that mimics the structure and function of the adult chicken gut microbiome, we performed culturomics by isolating strains that were representative of the bacterial phylum-level diversity typically found in a conventional chicken gut microbiome. From about 4000 colonies picked, 243 different species covering 88 genera across four phyla were obtained (Table S3). Ten strains, representing the most prevalent and abundant phyla, were selected for inclusion in a synthetic microbial community, referred to as SynCom^Bac10^ (Fig. 2A and Table S4). By interrogating 799 public chicken gut metagenomes and 585 chicken gut 16S rRNA gene amplicon sequencing samples, we demonstrated that most of the 10 bacterial species were ubiquitous in the chicken gut (Table S5): the top prevent species were *Limosilactobacillus reuteri* (with an average prevalence of 58.8%), followed by *Enterococcus faecium* (44.9%), and *Bifidobacterium pullorum* (31.3%). SynCom^Bac10^ members were predicted to carry 30 276 coding sequences (CDSs) belonging to 17 644 Kyoto Encyclopedia of Genes and Genomes Orthology (KOs) (Table S4). Visualizing the extent of overlap between KOs across various phylum levels within SynCom^Bac10^ revealed that common functions accounted for 9%, and unique KOs were 29%, 25%, 2%, 2%, and below 1% for *Firmicutes*, *Proteobacteria*, *Bacteroidota*, *Actinobacteria*, and *Verrucomicrobia*, respectively (Fig. 2B). SynCom^Bac10^ covered 70.5%, 86.4%, 88.2%, and 79.6% of the KOs detected in the adult microbial community of duodenum, jejunum, ileum, and cecum (Fig. 2C), respectively, suggesting the high functional coverage of SynCom^Bac10^ to adult chicken microbiota.

Each individual member of SynCom^Bac10^ exhibited normal growth in the Gifu Anaerobic Medium (GAM) liquid medium, except that *A. muciniphila* ATCC BAA-835 displayed a slow growth speed (Fig. S1A-J), whereas the influence of individual strains on the growth of other members in pairwise co-culture systems differed a lot (Fig. 2D). The majority of pairwise co-cultivated strains exhibited a neutral relationship (0/0, 26 of 45 of interactions), followed by commensalism (+/0 or 0/+, 10 of 45 of interactions) and amensalism (−/0 or 0/−, 7 of 45 of interactions); *M. funiformis* CML154 showed mutualistic interactions (+/+) with both *A. caccae* CML199 and *B. xylanisolvens* CML384. No competition (−/−) or predation (+/−) relationships were observed among the SynCom^Bac10^ members. All the SynCom^Bac10^ members could coexist when co-cultured together (Fig. 2E), and SynCom^Bac10^ was even stable when it was challenged by pathogens of *Salmonella typhimurium*, *E. coli*, or *Clostridium perfringens* (Fig. S2A–D).

### SynCom^Bac10^ modulates chicken physiological metabolism and promotes maturation of intestinal epithelial barrier

**Figure 3:**
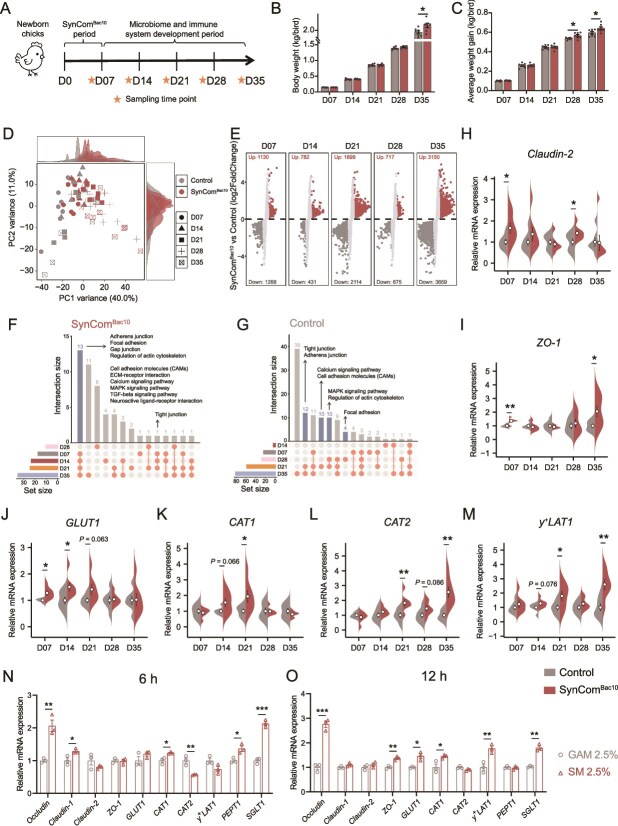
SynCom^Bac10^ changes physiological metabolism and promotes gut epithelial barrier maturation of chickens. (A) Schematic of SynCom^Bac10^ feeding experiment. (B) Body weight of chickens from day 7 to day 35. (C) Average weight gain of chickens from day 7 to day 35. (D) Principal component analysis (PCA) plot of expression levels from all genes between the control and SynCom^Bac10^ chickens from day 7 to day 35. (E) Differentially expressed genes (DEGs) in the control and SynCom^Bac10^ chickens. The UpSet plot highlights the intersection of KEGG pathways in the SynCom^Bac10^ (F) and control (G) chickens among the five sampling times. The relative mRNA expression of *Claudin-2* (H), *ZO-1* (I), *GLUT1* (J), *CAT1* (K), *CAT2* (L), and *y^+^LAT1* (M) between the two groups in the ileum. The relative mRNA expression of intestinal barrier proteins and nutrient transporters in IECs cultures containing 2.5% sterile fermentation supernatant of SynCom^Bac10^ at 6 h (N) and 12 h (O). ^*^  *P* value < .05, ^**^  *P* value < .01, and ^***^  *P* value < .001.

To reveal the role of SynCom^Bac10^ in vivo, early life chicken gut microbiome manipulation using SynCom^Bac10^ was performed (Fig. 3A). After a 7-day intervention of the newly born chicks, SynCom^Bac10^ significantly increased the chicken body weight at 35 days of age (*P* value < .05; Fig. 3B). Compared with the control group, SynCom^Bac10^ increased the chicken average weight gain and tended to decrease the feed-gain ratio in periods of days 21–28 and 28–35 (Fig. 3C and Fig. S3A), but did not affect the 35-day average feed intake (*P* value > .05; Fig. S3B). SynCom^Bac10^ had no obvious effect on the relative length of chicken duodenum, jejunum, and cecum during the whole growth period (*P* value > .05; Fig. S3C–E). However, it significantly increased the relative length of the ileum at day 7 and day 14 (*P* value < .05; Fig. S3F), which may enhance the host’s nutrient absorption during these stages.

We compared the transcriptome profiles of ileum tissue at the five sampling times between the two groups and found that SynCom^Bac10^ affected the ileum gene expression (Fig. 3D and E). A total of 13 differential Kyoto Encyclopedia of Genes and Genomes (KEGG) enrichment pathways were shared among four times (D07, D14, D21, and D35) in the SynCom^Bac10^ group (Fig. 3F). Besides, the tight junction pathway was enriched in the SynCom^Bac10^ group at D07, D14, and D21. In contrast, in the control group, genes enriched in the same pathways, including adherens junction, tight junction, focal adhesion, regulation of actin cytoskeleton, cell adhesion molecules (CAMs), calcium signaling pathway, and MAPK signaling pathway, were found to start from D21 (Fig. 3G). Because the majority of these pathways are related to intestinal mechanical barrier function, these results suggest a pre-maturation of the intestinal barrier after SynCom^Bac10^ intervention.

We measured the expression of genes related to intestinal barrier as well as nutrient transporters in the ileum. The relative mRNA expression levels of *Claudin-2* and zonula occludens-1 (*ZO-1*), which involved in maintaining the integrity of intestinal barrier, were higher at D07 in the SynCom^Bac10^-inoculated chickens than those in the control chickens (*P* value < .05; Fig. 3H and I), and the expression of the nutrient transport-related genes such as glucose transporter protein type 1 (*GLUT1*), cationic amino acid transporters (*CAT1* and *CAT2*), and y^+^ L amino acid transporter-1 (*y^+^LAT1*) were also significantly increased in different growth stages of SynCom^Bac10^ chickens (*P* value < .05; Fig. 3J–M). To further validate the effect of SynCom^Bac10^ on gut function, we isolated primary chicken embryo IECs to confirm the findings *in vitro*. Compared to the GAM control, the sterile spent culture medium (SM) of SynCom^Bac10^, at a concentration of 2.5%, significantly enhanced the relative mRNA expression levels of *Occludin*, *Claudin-1*, *ZO-1*, *GLUT1*, *CAT1*, *y^+^LAT1*, *PEPT1*, and *SGLT1* in IECs in either 6 hours or 12 hours (*P* value < .05; Fig. 3N and O). Similarly, the expression levels of *Occludin*, *Claudin-1*, *Claudin-2*, *GLUT1*, and *y^+^LAT1* were up-regulated when 5% SM was used (*P* value < .05; Fig. S4A and B).

Comparison of serum metabolic alterations at D35 between the control and SynCom^Bac10^ chickens revealed that SynCom^Bac10^ administration affected the chicken metabolic profiles (Fig. S5A and B). In the control group, glycerophospholipids constituted the majority of significantly changed metabolites (SCMs) (*n* = 37, 24.3%), whereas in the SynCom^Bac10^ group, SCMs were predominantly amino acids and their metabolites (*n* = 26, 18.8%) (Fig. S5C). Metabolic pathways significantly up-regulated in the SynCom^Bac10^ chickens included tyrosine metabolism, alanine, aspartate, and glutamate metabolism, as well as valine, leucine, and isoleucine degradation. Conversely, glycerophospholipid and glycerolipid metabolism was significantly down-regulated in the SynCom^Bac10^ group (*P* value < .05, Fig. S5D). These results indicate that SynCom^Bac10^ modulates the chicken physiological metabolism, particularly amino acid metabolism.

### Early life SynCom^Bac10^ exposure accelerates chicken gut microbiota maturation

**Figure 4:**
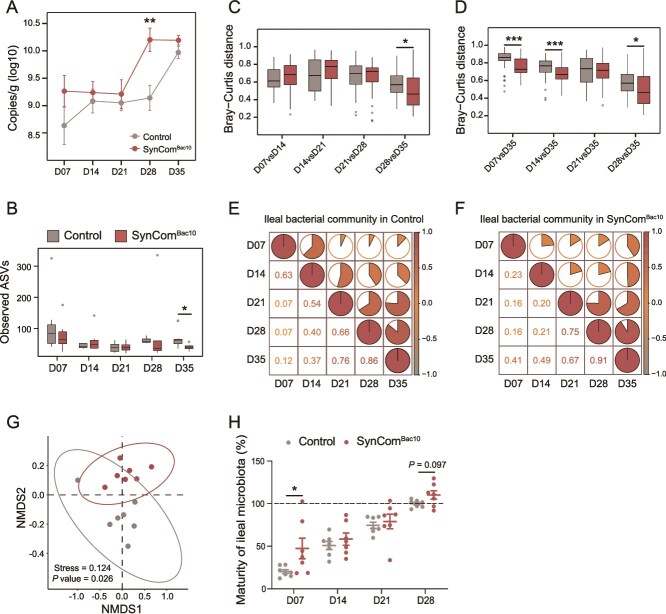
SynCom^Bac10^ accelerates ileal microbiota maturation. (A) Dynamics of bacteria in the ileum of the control and SynCom^Bac10^ chickens based on microbial absolute abundance. (B) Observed ASVs of ileal microbiota between the control and SynCom^Bac10^ chickens during 35 days. (C) Pairwise Bray-Curtis distances of ileal bacteria from adjacent sampling time points. (D) Bray-Curtis distances between the ileal bacteria at D35 and the other four sampling times. The pairwise correlations of bacterial communities between the control (E) and SynCom^Bac10^ (F) chickens at different sampling times, as reflected by Pearson’s correlation coefficients. (G) Non-metric multidimensional scaling (NMDS) plot based on Bray-Curtis distance of ileal microbiota composition between the control and SynCom^Bac10^ chickens at D07. (H) Maturity of ileal microbiota between the control and SynCom^Bac10^ groups. ^*^  *P* value < .05, ^**^  *P* value < .01, and ^***^  *P* value < .001.

To investigate the temporal dynamics of the chicken gut microbiota after SynCom^Bac10^ exposure, we analyzed the bacterial succession in both ileal and cecal content samples using relative microbiome profiling and quantitative microbiome profiling methods throughout the 35-day growth period. The results showed that the ileum of chickens in the SynCom^Bac10^ group harbored a higher load of bacteria than that in the control group, especially at D28 (*P* value < .05, Fig. 4A), whereas there were no significant alterations in cecal bacterial loads between the two groups (Fig. S6A). There were no differences in the α-diversities in both the ileal and cecal microbiota during the chicken growth between the two groups, except that, at D35, the ileum of SynCom^Bac10^ chickens showed a lower Observed ASVs (Figs 4B andS6B).

Comparison of the pairwise Bray-Curtis distances (β-diversity) at adjacent time points in the two group of chickens revealed that SynCom^Bac10^ accelerated changes of ileal bacterial communities within the first 28 days, leading to overall composition of bacterial communities in the SynCom^Bac10^ chickens at D28 and D35 more similar to each other (a lower distance) compared to that in the control group (*P* value < .05, Fig. 4C). Furthermore, the Bray-Curtis distances between the ileal microbiota of D35 and each of the other four sampling times decreased as the chicken grew, and the microbiota in D07, D14, and D28 were more similar to the microbiota in D35 in the SynCom^Bac10^ chickens than that in the control chickens (*P* value < .05, Fig. 4D). Similar trends were also observed in the cecal microbiota (Fig. S6C and D). The ileal microbiota appeared to develop in a step-wise manner during the chicken growth period. Seven-day-old chickens exhibited a microbiome community structure similar to that at 14 days, but this composition diverged significantly at 21, 28, and 35 days (Fig. 4E). SynCom^Bac10^ provoked a shift in the ileal bacterial structure of 7-day-old chickens, resulting in a community more similar to that in older chickens (Fig. 4F and G).

By combining the results of the principal coordinate analysis (PCoA) for both the ileum and cecum in the control chickens (Fig. S7A and B), we concluded that our sampling time points represented three different stages of the development of the chicken intestinal microbiota: juvenile phase (D07), transitional phase (D14 and D21), and mature phase (D28 and D35). To validate the effect of SynCom^Bac10^ on gut microbiota, we defined the mature phase of 28-day-old as 100% maturity, and random forest regression was used to predict the microbial maturity. SynCom^Bac10^ was found to keep accelerating gut microbiota development from D07, and prompted the microbiota to reach a mature state in advance (Fig. 4H). Simultaneously, whether in the ileum or the cecum, SynCom^Bac10^ had the most prominent effect on accelerating the maturation of the microbial community during the juvenile phase (*P* value < .05, Fig. 4H andS6E).

### SynCom^Bac10^ induces SFB pre-colonization in the chicken intestine

**Figure 5:**
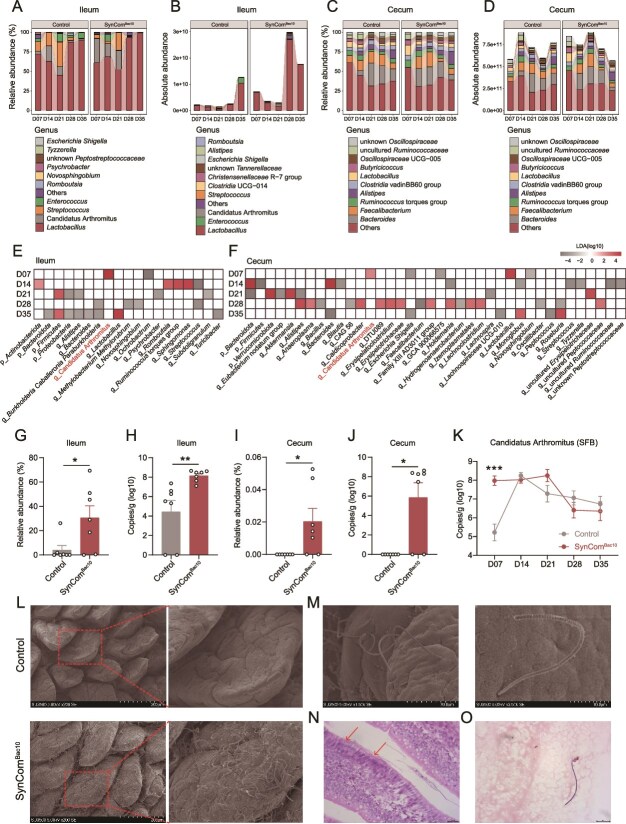
The dynamic development of gut microbiota in chickens after SynCom^Bac10^ exposure. Relative abundance (A) and absolute abundance (B) of ileal microbiota in the control and SynCom^Bac10^ chickens at the genus level. Relative abundance (C) and absolute abundance (D) of cecal microbiota in the control and SynCom^Bac10^ chickens at the genus level. Differentially abundant taxa were tested in ileum (E) and cecum (F) by Lefse with LDA score > 2 and *P* value < .05. Relative abundance (G) and absolute abundance (H) of SFB in the ileum at D07. Relative abundance (I) and absolute abundance (J) of SFB in the cecum at D07. (K) the abundance of SFB determined by RT-qPCR in the terminal ileum of the control and SynCom^Bac10^ chickens. (L) SEM of terminal ileum of 7-day-old control and SynCom^Bac10^ chickens. SFB morphology visualized by SEM (M), HE staining (N), and Gram staining (O). ^*^  *P* value < .05, ^**^  *P* value < .01, and ^***^  *P* value < .001.

To investigate the microbial changes that lead to accelerated maturation of the gut microbiota, we compared the microbial community composition of ileum and cecum between the control and SynCom^Bac10^ chickens. At the phylum level, *Firmicutes* predominated the ileal microbial community throughout chicken growth phase (Fig. S8A and B). In 7-day-old chickens, the cecal microbiota mainly consisted of *Firmicutes*, while from D14, the *Bacteroidota* began to expand (Fig. S8C and D). At the genus level, the *Lactobacillus* and *Candidatus* Arthromitus (segmented filamentous bacterium; SFB) dominated the ileum from D07 to D21 (Fig. 5A and B). The cecal bacterial community exhibited a more complex composition, with genera such as *Bacteroides*, *Faecalibacterium*, *Ruminococcus torques* group, and *Alistipes* predominating throughout the 35-day period (Fig. 5C and D). After SynCom^Bac10^ administration, seven out of the 10 SynCom^Bac10^ species can be found in either the chicken ileum or cecum across the different growth periods (Table S6).

The linear discriminant analysis effect size (LEfSe) analysis further demonstrated that SynCom^Bac10^ treatment significantly influenced the composition of both the ileal and cecal microbiota. *Candidatus* Arthromitus (SFB), *Lactobacillus*, *Streptococcus*, and *Novosphingobium* were common differential bacteria in both ileum and cecum between the two groups (Fig. 5E and F). Only *Candidatus* Arthromitus (SFB) exhibited significantly higher abundance simultaneously in both the ileum and cecum of the SynCom^Bac10^ group than the control group at D07 (*P* value < .05, Fig. 5G–J), suggesting that SynCom^Bac10^ promoted the early colonization of SFB in the chicken gut. A further detection of the dynamic changes of SFB in ileum during the whole chicken growth period indicated that the abundance of SFB showed a trend of increasing first and then decreasing in both groups of chickens (Fig. 5K). The SFB abundance was nearly three orders of magnitude higher in SynCom^Bac10^ chickens than that in the control chickens at D07 (*P* value < .001). This was further evidenced by the SEM result that the terminal ileum of 7-day-old SynCom^Bac10^ chickens was densely populated by SFB (Fig. 5L). SFB in the chicken ileum exhibited Gram-positive, long, filamentous morphology, with segments divided by discrete septa, which could even be observed in the section of SynCom^Bac10^ chicken ileal tissue under an optical microscope (Fig. 5M–O).

### Chicken immune responses triggered by the SynCom^Bac10^-induced SFB

As we have shown above, SynCom^Bac10^ induced the pre-colonization of SFB in the chicken gut at D07 (Fig. 5G–K), and SFB are known as potent inducers of Th17 cells in many animal species and in humans [27]. Therefore, we investigated whether the SFB stimulated by SynCom^Bac10^ could induce the proliferation of Th17 cells and cytokine production. The results revealed that Th17 cells were significantly accumulated in the ileum of SynCom^Bac10^ chickens (Fig. S9A and C), whereas no significant abundance differences were observed for Treg cells between the two groups at D07 (Fig. S9B and C). The induction of Th17 cells is substantiated by the upregulation of various Th17 effector cytokines including *IL-17A*, *IL-17F*, and *IL-22* [5], which was in accordance with our results in the SynCom^Bac10^ chickens (Fig. S9D–F). Moreover, compared with the control group, SynCom^Bac10^ considerably increased the gene expression of *IL-10* at D07, *IFN-γ* at D21, *TGF-β* at D35, and chemokine *CCL-17* at D07 and D14, whereas significantly decreased the expression of *IL-1β* at D14, D21, and D28 (Fig. S9G–K); a decreasing trend of gene expression of *iNOS* was also observed at D21 upon SynCom^Bac10^ treatment (Fig. S9L). Besides, at D07 and D28, serum IgA concentrations were significantly higher in SynCom^Bac10^ chickens compared to the control chickens (Fig. S9M), whereas serum IgG concentrations in SynCom^Bac10^ chickens were significantly lower than those in the control chickens (Fig. S9N), indicating a more mature immune system [28].

**Figure 6:**
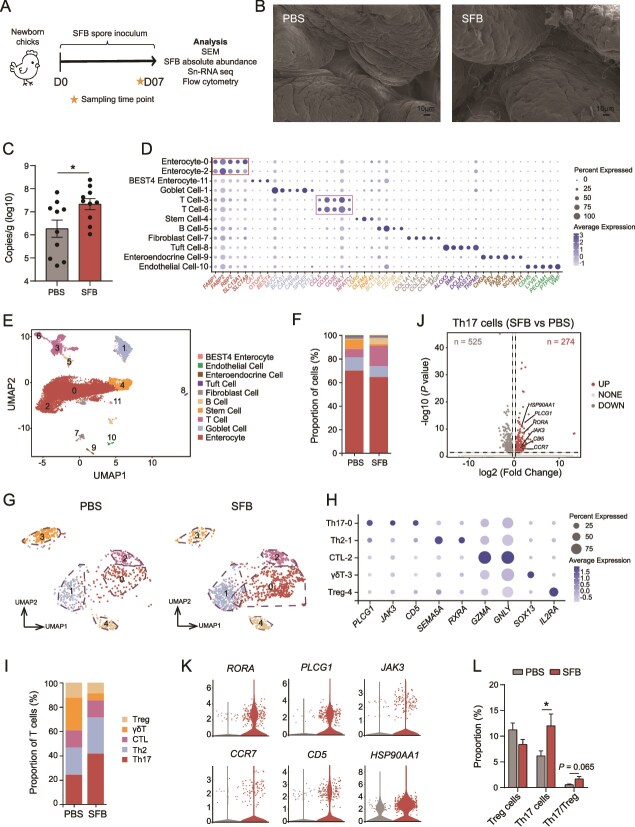
SFB spore triggers immune shifts in chicken gut. (A) Schematic of SFB spore feeding experiment. (B) SEM of terminal ileum of seven-day PBS and SFB chickens. (C) Absolute abundance of SFB in the ileum at D07. (D) Bubble plot illustrating the representative marker genes for different cell clusters. The dot size indicates the percentage of each cluster expressing the gene, and the color saturation reflects the average expression level. (E) Uniform manifold approximation and projection (UMAP) plot with 12 distinct cell clusters of 10 cell types based on snRNA-seq in the chicken ileal mucosa. The dot colors represent different cell types. (F) Bar chart showing the proportions of the 10 cell types. (G) UMAP plot with five T cell types between the PBS and SFB chickens. (H) Bubble plot illustrating the representative marker genes for different T cell clusters. (I) Bar chart showing the proportions of the five T cell types. (J) Volcano map of DEGs in Th17 cells of the PBS and SFB chickens. (K) Violin plot showing the expression levels of key DEGs related to Th17 cell differentiation. (L) the proportions of Th17, Treg cells, and their ratio in ileal lamina propria detected by flow cytometry.

To confirm the role of SynCom^Bac10^-induced SFB in modulating the chicken immune system, we inoculated the SynCom^Bac10^ chicken-born SFB spore into newborn chicks (Fig. 6A). We found that, after a 7-day inoculation, SFB filaments began to establish in the distal ileum of the receiver chicks, whereas they were absent in the PBS-treated chicks (Fig. 6B). The RT-qPCR results further confirmed that SFB spore inoculation markedly increased the number of SFB copies in the gut contents of the 7-day-old chickens (*P* value < .05, Fig. 6C). We then investigated the chicken ileal cell landscape after SFB spore intervention using single-nucleus RNA sequencing (snRNA-seq) of the mucosal samples. We discerned 12 cellular clusters that correspond to 10 unique cell types, based on the expression of cell-type-specific marker genes in the intestine (Fig. 6D and E). The proportions of immune cells (T cells and B cells) were found obviously increased in SFB chicken compared to the control (Fig. 6F). Among the five classified T cell types, SFB spore inoculation markedly increased both the absolute and relative number of Th17 cells, whereas it decreased the number of γδT cells (Fig. 6G–I). Compared with the PBS group, 274 genes in the Th17 cells of the SFB-colonized chicken were upregulated, and 525 genes were downregulated (Fig. 6J). Six genes related to Th17 cell differentiation, including *RORA*, *PLCG1*, *JAK3*, *CCR7*, *CD5*, and *HSP90AA1*, were all found upregulated with SFB spore treatment (Fig. 6K), highlighting the substantial role of SFB in inducing Th17 cells. The induction of Th17 cells after SFB spore inoculation was also evidenced by the flow cytometry results (Fig. 6L).

### SynCom^Bac10^ protects chickens from *Salmonella* infection

**Figure 7:**
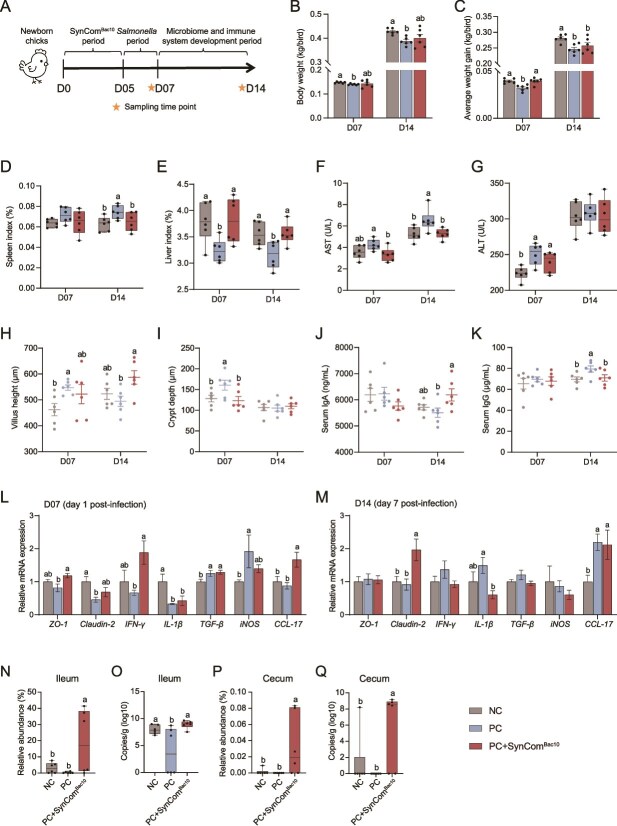
SynCom^Bac10^ chickens resists *Salmonella* infection. (A) Schematic of *Salmonella* infection experiment. (B) Body weight of chickens. (C) Average weight gain of chickens. (D) Spleen index. (E) Liver index. (F) Serum AST levels. (G) Serum ALT levels. (H) Ileal villus height. (I) Ileal crypt depth. (J) Serum IgA concentration. (K) Serum IgG concentration. The relative mRNA expression of tight junction proteins and immunity-related genes in the ileum at D07 (L) and D14 (M). Relative abundance (N) and absolute abundance (O) of SFB in the ileum at D07. Relative abundance (P) and absolute abundance (Q) of SFB in the cecum at D07.

To determine whether SynCom^Bac10^ administration can resist *Salmonella* infection in chicken gut, we performed a *Salmonella* challenge experiment (Fig. 7A). *Salmonella* infection reduced the chicken body weight and average weight gain (Fig. 7B and C), increased the spleen index (Fig. 7D), decreased the liver index (Fig. 7E), elevated the serum levels of organ damage indicators AST and ALT (Fig. 7 F and G), and affected the ileal villus height and crypt depth (Fig. 7H and I). SynCom^Bac10^ significantly improved these phenotypic indexes at day 1 or day 7 post-infection (Fig. 7B–I). At day 7 post-infection, the serum IgA concentration was significantly higher in the PC + SynCom^Bac10^ chickens compared to the PC chickens (Fig. 7J), whereas the IgG concentration was significantly lower in the PC + SynCom^Bac10^ group compared with PC chickens and similar to NC chickens (Fig. 7K). SynCom^Bac10^ also increased the gene expression of *ZO-1*, *IFN-*γ, and *CCL-17* at day 1 post-infection (Fig. 7L), and increased *claudin-2* and *CCL-17*, yet decreased *IL-1β* expression at day 7 post-infection (Fig. 7M). In agreement with the aforementioned findings, SynCom^Bac10^ significantly increased the abundance of SFB in the ileum and cecum at D07 in PC + SynCom^Bac10^ chickens (Fig. 7N–Q). Given the above results that SynCom^Bac10^-induced SFB triggered Th17 cell-mediated immune responses, the observed enrichment of SFB in the chicken gut likely contributed to the positive effect of SynCom^Bac10^ in resisting *Salmonella* infection.

### Assembling SFB genomes from the SynCom^Bac10^ chickens

**Figure 8:**
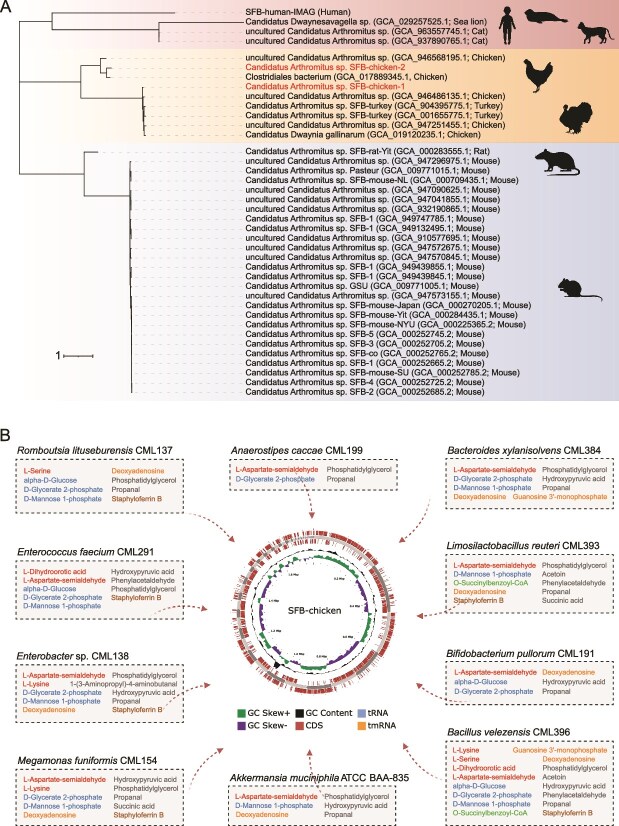
Phylogenetic analysis and cross-feeding prediction. (A) SNPs-based phylogenetic tree of 39 SFB genomes, including a human, a rat, 25 from mice, two from turkeys, seven from chickens, two from cats, and a sea lion. Scale bar: 1 indicates that each unit length on the phylogenetic tree corresponds to 1 SNP genetic variation. (B) the potentially transferable metabolites from SynCom^Bac10^ to SFB. SFB (genome map) serves as a metabolite taker, the 10 SynCom^Bac10^ members (large rectangles) serve as metabolite givers, and the arrows illustrate metabolite transfer.

As SynCom^Bac10^ induced the proliferation of SFB, we tried to assemble the genomes of these difficult-to-culture bacteria from the chicken gut. Two metagenomic bins of SFB were reconstructed from the sequencing data of the 7-day-old SynCom^Bac10^ chicken terminal ileum samples. One genome, hereafter referred to as *Candidatus* Arthromitus sp. SFB-chicken-1 comprised 1 759 061 bp (54 contigs) with completeness of 94.8%. The second genome, designated as *Candidatus* Arthromitus sp. SFB-chicken-2, covered 1 572 202 bp (197 contigs) and possessed 86.5% completeness. The relative abundances of SFB-chicken-1 and SFB-chicken-2 were determined to be 20.3% and 2.8%, respectively. The reconstructed genomes were subjected to single-nucleotide polymorphisms (SNPs)-based phylogenetic analysis with SFB from different host species (Fig. 8A and Table S7). The first phylogenetic branch comprises SFB strains from humans, cats, and sea lions. The second branch mainly includes SFB strains from chickens and turkeys, with the two chicken SFB genomes we assembled distributed among two distinct subclusters. The third branch encompasses SFB strains isolated from mice and rats, where the rat-derived SFB strains are relatively distant from those of mice origin. We then compared the average amino acid identity (AAI) and average nucleotide identity (ANI) among the 39 SFB genomes (Fig. S10A and B). The results indicated that all SFB from different hosts belong to the same genus but represent distant species. Two different SFB species were identified from the chickens: SFB-chicken-1 and SFB-chicken-2, exhibiting 76.3% AAI and 83.2% ANI, each belonged to a distinct species.

### SynCom^Bac10^ potentially provides essential metabolites for SFB growth

To investigate the genome differences of different SFB strains, a comparative genome analysis was performed utilizing eight representative SFB strains isolated from different hosts (Table S8). The results revealed a conserved set of metabolic capabilities and deficiencies in SFB strains. Consistent with their biological features, SFB genome showed characteristics of typical anaerobic organisms, including a nearly complete repertoire of enzymes for anaerobic glycolysis and fermentation, whereas lacking components of the tricarboxylic acid cycle and respiratory chain-related proteins (Table S9). A more striking metabolic characteristic of SFB was the almost lack of genes involved in the biosynthesis of amino acids, vitamins/cofactors, carbohydrates, and nucleotides (Fig. S11A–D). Comparison of the metabolic pathways between the SynCom^Bac10^ members and SFB revealed that SynCom^Bac10^ seemed to largely compensate for the missing metabolic potentials in SFB (Fig. S11A–D).

As we showed that SynCom^Bac10^ stimulated the pre-colonization of SFB in chicken gut (Fig. 5G–K), we speculated that early inoculation of SynCom^Bac10^ probably provided nutrients that promoted the growth of SFB through metabolite cross-feeding. To validate this, we predicted the potentially transferable metabolites from SynCom^Bac10^ members to SFB using iNAP 2.0 [29]. This analysis revealed a total of 36 putative transferable metabolites derived from different SynCom^Bac10^ members (Table S10). To verify whether these metabolites could indeed be produced by the SynCom^Bac10^ strains, we performed metabolomic profiling of the bacterial culture supernatants, and ultimately, a total of 18 metabolites were confirmed (Fig. 8B and Table S11). *B. velezensis* CML396 was identified as the strain providing the largest number of transferable metabolites, mainly amino acids and carbohydrates. As for metabolites, coenzyme A derivatives (such as o-succinylbenzoyl-CoA) and nucleosides, nucleotides, and analogs (e.g., deoxyadenosine and guanosine 3′-monophosphate) were found to be transferable from different members to SFB. The genomes of SFB encode iron transporter systems that facilitate the uptake of siderophores (Table S9), which was one of the ways for bacterial iron uptake. We found that staphyloferrin B, a siderophore, could be provided from six SynCom^Bac10^ members (*L. reuteri* CML393, *B. velezensis* CML396, *E. faecium* CML291, *M. funiformis* CML154, *R. lituseburensis* CML137, and *E.* sp. CML138) to SFB.

## Discussion

In this study, we constructed a synthetic microbial community (SynCom^Bac10^) comprising 10 bacterial strains that represented five bacterial phyla commonly found in the chicken gut. We demonstrated that SynCom^Bac10^ covered ~70%–88% of the microbial functional categories presented in four different segments of the chicken gut. Apart from considering the phylogenetic and functional representation of SynCom^Bac10^, additional reasons for the strain selection are as follows: (i) *L. reuteri* CML393 and *B. velezensis* CML396 were verified to be beneficial for chicken growth in our previous study [30]; (ii) *E. faecium* CML291 and *E.* sp. CML138 are facultative anaerobes that might facilitate the establishment of strict anaerobes [23]; (iii) *M. funiformis* CML154, *R. lituseburensis* CML137, and *B. xylanisolvens* CML384 are immunomodulatory microorganisms that contributes to host immune system development and resistance to pathogen infections [13]; and (iv) three strains belonging to the potential keystone species reported previously [31], were also added to the SynCom^Bac10^ consortium, i.e., *A. caccae* CML199, *B. pullorum* CML191, and a commercial strain, *A. muciniphila* ATCC BAA-835. We also showed that SynCom^Bac10^ met the standards for designing a synthetic microbial community [24]. Each community member has favorable growth behavior, and the interactions of members do not involve growth-limiting behaviors such as competition and predation. Additionally, all members of the community can stably coexist. SynCom^Bac10^, thus, represents a satisfactory synthetic community with functionally well-defined strains included in and having acceptable coverage of natural community structure and function.

The composition of the chicken gut microbiota is dynamic, undergoing successive transitions prior to the establishment of a mature community [9, 32]. In the present study, we showed that SynCom^Bac10^ accelerated the chicken gut microbiota development, and eventually led to the microbiota to mature in advance. SynCom^Bac10^, chickens with a pre-mature gut microbiota in this study, displayed an improvement of gut health, better barrier, and immune function, and a resulting better growth performance. Similar findings have also been found in studies aiming to manipulate chicken gut microbiota at early life, such as inoculating adult-derived microbiota into newborn chicks or fertile eggs, and keeping newly hatched chicks in contact with adult hens [33–35]. The maturation of gut microbiota can be determined not only by the community structure but also by the abundance of indicator taxa. *Lactobacillus* has been demonstrated to facilitate the development and maturation of intestinal microbiota in chickens and piglets [36–38], and *Streptococcus* and *Novosphingobium* measured the balance of intestinal microbial community, which gradually decreased as the intestinal microbiota matured [39, 40]. Here, we found a significantly higher abundance of *Lactobacillus* and a decreased abundance of *Streptococcus* and *Novosphingobium* in the SynCom^Bac10^ chickens, further indicating a more stable and mature microbial community after SynCom^Bac10^ intervention.

Another feature regarding the mature chicken microbiota was the early appearance of SFB in the chicken gut. SFB has been widely confirmed to play an indispensable role in the development of host intestinal immune barrier by triggering both innate and adaptive immune responses. Particularly, SFB are potent inducers of intestinal Th17 cells [5]. In consistent with this, we showed that in the SFB-enriched SynCom^Bac10^ chickens, the proportions of Th17 cells were significantly increased, with a resulting increase of the effector cytokines including *IL-17A*, *IL-17F*, and *IL-22*. The induction of Th17 cells was further evidenced by the inoculation of SynCom^Bac10^ chicken SFB spores into newborn chicks, and critical genes related to Th17 cell differentiation in the SFB-treated chickens were identified through snRNA-seq analysis. Mechanisms underlying SFB-induced Th17 cell differentiation are complex and multifaceted. For example, SFB enhances retinoic acid (RA) levels and activates retinoic acid receptor (RAR) signaling in intestinal epithelial cells, which promotes innate epithelial defense and drives Th17 cell differentiation [41, 42]. Moreover, the tight adherence of SFB to intestinal epithelial cells may trigger the expression of serum amyloid A, which activates dendritic cells to produce *IL-23*, thereby establishing a cytokine environment conducive to Th17 cell development in the intestine [5]. Additionally, SFB-induced changes in gut microbiota composition could indirectly modulate the immune response.

Studies have demonstrated that the immunity induced by SFB reduced the infection of *S. typhimurium* and *E. coli* [43, 44]. In accordance with these findings, we verified that the SFB-enriched SynCom^Bac10^ chickens were highly resistant to *Salmonella* infection due to the SFB-mediated immune responses. It is reported that *IL-17A*, *IL-17F*, and *IL-22* secreted by Th17 cells help the host defend against both bacterial and fungal infections, especially at the mucosal surfaces [5]. Moreover, SFB can induce humoral immune, characterized by the elevated levels of both mucosal and serum IgA [45]. IgA-mediated intracellular killing via phagocytosis is highlighted in different invasive pathogenic bacteria, including *Streptococcus pneumoniae*, *E. coli*, and *Staphylococcus aureus* [46]. Additionally, we found a significantly elevated gene expression of *IFN-γ* in the ileum of the PC + SynCom^Bac10^ chickens. *IFN-γ*, an immunostimulatory cytokine produced by T lymphocytes and natural killer cells, was reported to be associated with enhanced T-cell responses and rapid *Salmonella* clearance in chickens [47].

Although SFB-mediated immune responses played a crucial role in the clearance of *Salmonella* infection in this study, we need to point out that non-immune factors cannot be excluded. First, the pre-colonization of SFB may tightly adhere to ileal epithelium in advance, and thus reduce the colonization niches for *Salmonella* [48]. Second, a strengthening of the intestinal epithelial barrier could resist the pathogen invasion. This barrier enhancement can be attributed to either the SynCom^Bac10^ per se in promoting barrier-related gene expression, as we evidenced in the cell experiment, or the positive role of the Th17-derived cytokines *IL-17* and *IL-22* in enhancing and regulating tight junctions and mucin synthesis [49]. Third, a recent study indicated that SFB promoted early protection against *Citrobacter rodentium* infection independent of CD4^+^ T cells, but relying on the production of RA [42]. Whether or to what extent RA produced by SFB helps the chickens to defend against *Salmonella* infection in this study warrants further investigation. We should finally emphasize that the anti-*Salmonella* effect of SynCom^Bac10^ treatment may not be solely attributed to its induction of SFB colonization. Members of SynCom^Bac10^ may directly contribute to this effect through the production of antimicrobial compounds, resource competition, or modulation of the gut environment.

SFB are typical commensal bacteria with strong auxotrophic needs that place them between obligate and facultative symbionts [50]. Although co-culturing SFB with epithelial cells has been reported [51], they are still uncultivable as isolates [52–54]. Here, by metagenomic sequencing method, we assembled two draft genomes of SFB (SFB-chicken-1 and SFB-chicken-2) from the SynCom^Bac10^ chickens. We showed that SFB-chicken-1 was predominant in the 7-day-old chicken gut, with relative abundance even reaching 20.3%, whereas SFB-chicken-2 only accounted for 2.8% of the total microbial abundance, which probably suggests a high colonization ability of SFB-chicken-1. We found that SFB-chicken-1 and SFB-chicken-2 are two different bacterial species belonging to the same genus. Though studies have suggested that different SFB species might exist in the chicken gut based on 16S rRNA genes [55], no detailed species and their genome sequences have yet been definitely proposed. We further demonstrated that SFB of different host origins all belonged to the same bacterial genus, but different animal hosts had different SFB species, except that the same SFB species existed in both chickens and turkeys. We also showed that SFB species of different host origins differed in metabolic capacities, probably due to their adaptive evolution to accommodate the hosts. These results confirmed previous findings regarding the host specificity of SFB, that SFB isolated from rat intestines are unable to colonize in the gut of mice or chickens [56, 57]. However, our study additionally suggests that host evolutionary relationships should be fully considered when interrogating the host specificity of SFB, and clear taxonomic assignments of SFB species are highly needed.

This study demonstrated that SynCom^Bac10^ promoted the pre-colonization and growth of SFB in the chicken gut. We hypothesized that this effect is attributable to cross-feeding interactions between SynCom^Bac10^ members and SFB, i.e., SynCom^Bac10^ members provided essential nutrients (cannot be self-sufficient) for SFB growth. This was supported by analyses of transferable metabolites from SynCom^Bac10^ members to SFB. Additionally, this hypothesis can be evidenced by the fact that SynCom^Bac10^ was more effective in promoting SFB colonization and proliferation than direct SFB spore inoculum (average SFB abundances: 10^8^ copies/g and 10^7^ copies/g at D07 for the two intervention manners, respectively). Apart from providing nutrients such as amino acids, carbohydrates, and nucleotides, six SynCom^Bac10^ members were found to transfer staphyloferrin B (a siderophore) to SFB. This result was consistent with previous reports that iron is essential for SFB growth [51], and SFB can capture iron from the environment through siderophores for acquisition of insoluble Fe^3+^. However, SFB lack the ability to synthesize siderophores, instead relying on the plentiful siderophores produced by neighboring species [50]. In addition to metabolite cross-feeding, the pre-colonization of SFB may be facilitated by the SynCom^Bac10^-induced modifications in the gut environment that establish favorable conditions for SFB growth, including altered local pH levels, oxygen concentration, and availability of substrates, which warrant further investigation.

The current study highlights the potential of SynCom^Bac10^ to improve intestinal homeostasis and anti-*Salmonella* immunity in chickens, yet it has several limitations. First, the specific colonization patterns of individual SynCom^Bac10^ members in the gut remain ambiguous, complicating the identification of strains directly responsible for the observed effects. Second, it remains to be determined whether a specific strain or strain combination, rather than the entire consortium, is sufficient to elicit these beneficial effects. Third, although the current SynCom^Bac10^ was designed based on phylogenetic diversity and functional representation, it may not represent the optimal strain combination for maximizing gut health and immune benefits. Future research should explore alternative strain combinations, including those with enhanced metabolic capabilities or stronger immunomodulatory properties, to further improve the efficacy of the synthetic community. Fourth, in this study, SynCom^Bac10^ was administered to the chicks via oral gavage, which limits its scalability for application in the poultry industry. Future delivery methods of SynCom^Bac10^, including incorporation into starter diets or drinking water, as well as application via innovative techniques such as *in ovo* injection [58], holds considerable promise for practical implementation.

In conclusion, we demonstrated here that a synthetic microbial community improved chicken intestinal homeostasis, especially protected the host against *Salmonella* infection via SFB-activated Th17 cell-mediated immune responses. The current work provides a paradigm for early life manipulation of the gut microbiota applying a designed microbial community to improve animal and potentially human gut health and infectious disease resistance.

## Supplementary Material

R3-ISMEminor-Supplementary_Materials_wraf076

R3-ISMEminor-Supplementary_Tables_wraf076

## Data Availability

All sequencing data and assembled genomes have been uploaded in the NCBI SRA database under the accession numbers PRJNA1211533 (microbiota data), PRJNA1211542 (RNA-seq data), and PRJNA1211720 (assembled genomes). All other relevant data are available in the supplementary materials.
