# Supplementary material for: Synthetic microbial community improves chicken intestinal homeostasis and provokes anti-Salmonella immunity mediated by segmented filamentous bacteria
Source: ISME J. 2025 Apr 23;19(1):wraf076. doi: 10.1093/ismejo/wraf076 (PMC12527353; doi:10.1093/ismejo/wraf076)
Supplement: R3-ISMEminor-Supplementary_Materials_wraf076 [file r3-ismeminor-supplementary_materials_wraf076.pdf]

Supplementary Materials

Synthetic microbial community improves chicken intestinal homeostasis and provokes anti-*Salmonella* immunity mediated by segmented filamentous bacteria

Short running title: SynCom improves chicken health

Meihong Zhang[1], Suxin Shi[1], Yimei Feng[1], Fengwenhui Zhang[1], Yuxuan Xiao[1], Xin Li[1], Xingliang Pan[2], Yuqing Feng[1], Dan Liu[1], Yuming Guo[1,*], Yongfei Hu[1,*]

[1]State Key Laboratory of Animal Nutrition and Feeding, College of Animal Science and Technology, China Agricultural University, Beijing 100193, China.
[2]Beijing General Station of Animal Husbandry, Beijing, 100107, China.

[*]Corresponding author:
Yongfei Hu, E-mail: huyongfei@cau.edu.cn. No. 2 Yuanmingyuan West Road, Haidian District, Beijing, 100193, China
Yuming Guo, E-mail: guoyum@cau.edu.cn. No. 2 Yuanmingyuan West Road, Haidian District, Beijing, 100193, China

The PDF file includes:

Supplementary Methods

Supplementary Figures S1 to S11

Supplementary Methods

Culturomics

The collected chicken intestinal contents were serially diluted using sterile phosphate buffered saline (PBS) under a super-clean bench. Each dilution was spread onto Luria-Bertani (LB), De Man, Rogosa and Sharpe (MRS), Gifu Anaerobic Medium (GAM), Brain Heart Infusion (BHI), and Fastidious Anaerobe Agar (FAA) plates, which were then incubated under aerobic conditions or anaerobic conditions (10% hydrogen, 10% carbon dioxide, and 80% nitrogen atmosphere) at 37°C for 1-7 days. Well-isolated colonies with different color and morphological shape were selected and purified on new plates. Amplification and sequencing analysis of the 16S rRNA genes were performed for each isolate, and all identified strains were preserved in corresponding growth medium with glycerol (20% v/v) at -80°C.

Bacterial growth measurement and pairwise co-culture assay

The bacterial growth was measured using 96-well deep well plates. The liquid volume in each well was 2 mL with 1.8 mL GAM culture medium and 200 μL inoculum. The growth of each strain was measured at OD_{600} every 3 h for 48 h using EPOCH2 Microplate Readers (BioTek). For pairwise co-culture, the PBS bacterial suspension was mixed with a 1:1 ratio. Then, 200 μL mixture was inoculated into the 96-well deep well plates as above. The outcomes of co-culture experiment were quantified through measuring the 16S rRNA gene copy numbers at the conclusion of triplicate co-culture experiments according to a previous study [1]. The interaction between two strains was categorized as either positive (+) or negative (-) if absolute abundance of a strain was significantly increased or decreased in co-culture relative to monoculture (P value < 0.05), respectively. In instances where the strain's abundance in co-culture

was statistically indistinguishable from its abundance in monoculture (*P* value > 0.05), the interaction was deemed neutral (0). Significance was determined using a t-test. Based on the microbial interactions (positive, negative, and neutral), there were six different types of interactions which could be categorized as follows: mutualism (+/+), commensalism (+/0), amensalism (−/0), predation (+/−), competition (−/−), and no interaction (0/0).

Community growth and pathogen interference assays

To monitor the bacterial growth in $\text{SynCom}^{\text{Bac10}}$, the ten $\text{SynCom}^{\text{Bac10}}$ members were mixed with an equivalent ratio and cultured in 48-well deep well plates. The culture volume was 5 mL containing 4.5 mL GAM medium and 0.5 mL bacterial mixture. Samples for RT-qPCR analysis were taken at 24 h and 48 h. For pathogen interference assay, the suspensions of *Salmonella* typhimurium, *Escherichia coli*, and *Clostridium perfringens* were prepared with an OD_{600} of 0.5, and each dilution was centrifuged at 6000 × g for 5 minutes. After centrifugation, the pellets were washed three times and resuspended in an equal volume of PBS for standby. The $\text{SynCom}^{\text{Bac10}}$ culture was prepared as described above. After a 12-hour culture period of $\text{SynCom}^{\text{Bac10}}$, pathogens were respectively inoculated, extending the total culture time to 48 hours. The OD_{600} was measured every 3 hours to monitor dynamic shifts in $\text{SynCom}^{\text{Bac10}}$ community.

$\text{SynCom}^{\text{Bac10}}$ administration experiment

One hundred and forty newborn Arbor Acres male chicks with similar body weight were randomly divided into either a control group or a $\text{SynCom}^{\text{Bac10}}$ group (7 replicates/group × 10 chicks/replicate) for 35 days. The seven control replicates and

the seven SynCom^{Bac10}-treated replicates were alternately placed in separate cages. All chickens were kept in cages under identical environmental conditions and provided with the same diets and water ad libitum. A total of 200 μL of sterile PBS (control group) or SynCom^{Bac10} in PBS (SynCom^{Bac10} group) was administered to each chick via oral gavage for seven consecutive days, starting from the first day of hatch. At each sampling time (D07, D14, D21, D28, and D35), seven chickens were randomly selected from each replicate, weighed, and euthanized using electro-anesthesia.

Salmonella challenge experiment

A total of ninety newly hatched Arbor Acres male chicks with similar body weight were randomly assigned to three groups: PBS control group (NC), *Salmonella* infection group (PC), SynCom^{Bac10} treatment and subsequently *Salmonella* infection group (PC+SynCom^{Bac10}), with six replicates per group and five chicks per replicate for 14 days. The chicks were gavaged with 200 μL PBS or SynCom^{Bac10} suspension from day 0 to day 4, and the five-day-old chicks were orally challenged with 1 mL PBS or *Salmonella* Typhimurium suspension at a concentration of 10^8 CFU/mL at day 5 and day 6. Chickens were weighed and euthanized using electro-anesthesia on day 7 (day 1 post-infection) and day 14 (day 7 post-infection) for sampling.

SFB spore preparation and administration

The methods for the enrichment of ileal SFB spores were according to previously described protocols with minor modifications [2, 3]. Briefly, scrapings from the terminal ileum of seven-day-old SynCom^{Bac10} chickens were pooled and resuspended in PBS containing 3 mM EDTA at a ratio of 10 times the sample (w/v), and then the suspension was treated with chloroform (3% of total solution, v/v) at room

temperature for 10 min. The use of chloroform not only promoted the formation of SFB spores but also ensured that the inoculum was free from other culturable microbes. The aqueous layer was filtered to new centrifuge tubes using a cell strainer (mesh size of 100 μm) and injected with nitrogen gas to remove residual chloroform from the spore preparations. The processed solution was centrifuged at 4500 × g for 15 min and the pellet was resuspended in a peptone-glycerol solution comprising 1% peptone (w/v) and 15% glycerol (v/v). The prepared suspension was cultured on GAM agar plates, and if necessary, subjected to gradient dilution to confirm the absence of growth from any culturable microbes. For SFB spore administration, twenty newborn Arbor Acres male chicks with similar body weight were randomly placed into two groups (n = 10 chicks/group). Each chick was orally inoculated with either 200 μL sterile chloroform-treated PBS (PBS group) or chloroform-treated ileal scrapings (SFB group) for 7 days. The SFB inoculum contained 10^5 SFB per 200 μL aliquot as quantified by RT-qPCR. Chickens were euthanized under electric shock anesthesia at day 7.

Isolation of primary chicken embryo intestinal epithelium cells and SynCom^{Bac10} treatment

Primary chicken embryo IECs were isolated from 17-day-old pathogen-free chicken embryonated eggs. IECs were cultivated in complete media containing DMEM/F12 (Gibco, USA), 2.5% fetal bovine serum (FBS), 1% penicillin-streptomycin-gentamicin solution, 20 ng/mL epidermal growth factor, 100 μg/mL heparin sodium, and 5 μg/mL insulin at 37°C with a humidified atmosphere containing 5% CO_2. After 48 hours of cells grew in 6-well culture plates, each well was washed three times with PBS, followed by the addition of 2 mL DMEM/F12

complete medium with or without the SynCom^{Bac10} member metabolic supernatant (2.5% or 5%) for periods of 6 hours and 12 hours. The IECs were subsequently washed with PBS and the intracellular RNA was extracted for measuring relative mRNA expression of tight junction proteins and nutrient transporters.

Whole-genome sequencing

All SynCom^{Bac10} members were sequenced using the HiSeq System (Illumina) served by the Institute of Microbiology of the Chinese Academy of Sciences (Beijing, China). Quality control and filtering of raw reads were performed using FastQC and Fastp, respectively. SPAdes was utilized to assemble clean reads into contigs and Prokka was employed for genome annotation. KOs were predicted by the BlastKOALA tool (https://www.kegg.jp/blastkoala/). The 16S rRNA gene amplicon sequencing data for duodenum, jejunum, ileum, and cecum of 35-day-old chickens was obtained from National Center for Biotechnology Information (NCBI) Sequence Read Archive (SRA) under accession number PRJNA817429, and PICRUSt2 was used to predict the KOs for functional comparisons with SynCom^{Bac10}. Public chicken gut metagenome (National Microbiology Data Center, https://nmdc.cn/icrggc/) and 16S rRNA gene amplicon sequencing (PRJNA1164933, PRJNA1086343, PRJNA1040402, PRJNA992971, PRJNA981342, PRJNA909494, PRJNA876288, PRJNA858221, PRJNA838439, PRJNA767330, PRJNA753623, PRJNA747060, PRJNA725811, PRJNA716565) were matched against SynCom^{Bac10} genomes and 16S rRNA gene sequences using sylph and blast, with stringent nucleotide identity thresholds of 95% and 97%, respectively. The species prevalence analysis was performed with protologger tool (https://protologger.bi.denbi.de/) [4].

DNA extraction and absolute real-time quantitative PCR

Bacterial DNA was extracted using Bacteria Genomic DNA Kit (CWBIO, Beijing, China) according to the manufacturer’s instructions. DNA concentration was subsequently determined by EPOCH2 Microplate Readers (BioTek) and checked by electrophoresis. A targeted DNA concentration was prepared by PCR amplification using DNA isolated from targeted microbial strain. All specific primers were synthesized by Sangon Biotech (Shanghai, China) and listed in Table S1. The PCR products were connected with the pEASY Blunt Cloning vector (TransGen Biotech, Beijing, China) and transformed into *Escherichia coli* DH5α (TransGen Biotech, Beijing, China) to generate standard plasmids. A 10-fold continuous dilution of plasmid DNA was added to the PCR plate to construct the standard curves for each individual strain, and the copy number of target gene was calculated using the following formula. All samples were analyzed by RT-qPCR in triplicate and the results were expressed as log10 copies of the gene per mL of bacterial fluid or per gram of content.

$$\text{DNA (copy)}=\frac{6.02\times 10^{23}\ \text{(copy/mol)}\times \text{DNA amount (g)}}{\text{DNA length (bp)}\times 660\ \text{(g/mol/bp)}}$$

16S rRNA gene amplicon sequencing and microbiome analysis

The QIAamp Fast DNA Stool Mini Kit (Qiagen, Germany) was used to extract the bacterial genomic DNA from the chicken ileal or cecal contents according to the manufacturer's instructions. Qualified DNA samples were sequenced targeting the V3-V4 variable region of the 16S rRNA genes. The paired-end sequencing was performed using NovaSeq System (Illumina) served by Majorbio Bio-pharm Technology Co., Ltd. (Shanghai, China). The raw sequencing data underwent quality control, denoising, merging, and amplicon sequence variant (ASV) identification

using the DADA2 plugin in the QIIME2 framework. Taxonomic classification of each ASV was achieved using a Naive Bayes classifier based on the SILVA database release 138. The absolute abundance was calculated by the following formula: estimated absolute abundance of taxon A = relative abundance of taxon A × gene copies number of total bacteria [5]. The α-diversity and β-diversity (Bray-Curtis distance) were calculated using the R package vegan. The microbial pairwise correlations at different time points were constructed using Pearson correlation coefficients and visualized using the corrplot package. Random forest regression, implemented using the R package randomForest, was employed to regress the taxa abundances in the temporal profiles of control gut microbiota against their chronological age according to the method described in a previous study [6]. The maturity index for the SynCom^{Bac10} group at a given time point was calculated using the random forest model with the control group serving as the training dataset, where the predicted time point represented the degree of maturity on the Y-axis and the actual time point was plotted on the X-axis. Differentially abundant taxa were identified by LEfSe with the LDA score > 2 and *P* value < 0.05.

Transcriptome sequencing analysis

Total RNA was extracted from ileal tissue that had been flash-frozen in liquid nitrogen utilizing TRIzol reagent (Beyotime, Shanghai, China). The integrity of RNA was assessed using an Agilent5300 Bioanalyzer, and purity and concentration were quantified with a Nanodrop2000 spectrophotometer (Thermo Scientific, USA). Construction and sequencing of the cDNA libraries were performed using NovaSeq System (Illumina) at Majorbio Bio-pharm Technology Co., Ltd. (Shanghai, China). Differentially expressed genes (DEGs) were identified using the DeSeq2 package in R

software and KEGG pathway enrichment analysis were conducted for these DEGs using the KOBAS (http://bioinfo.org/kobas/). The intersecting sets were analyzed using the R package UpSetR.

Metabolite measurement and analysis

Metabolites from serum or microbial supernatants were measured using untargeted metabolomics with a liquid chromatography-mass spectrometry system at Metware Biotechnology Co., Ltd (Wuhan, China). Metabolites were visualized using orthogonal partial least squares discriminant analysis (OPLS-DA). The SCMs were determined using a variable importance in projection (VIP) > 1 and P value < 0.05 with SIMCA software (Umetrics, Sweden). Metabolite classification was carried out by referencing the Human Metabolome Database (HMDB). Enriched KEGG pathways were explored by the MetaboAnalyst platform (http://www.metaboanalyst.ca).

Single-nucleus RNA sequencing

Frozen ileal mucosa was homogenized in a lysis buffer, filtered, and centrifuged to isolate nuclei, which were then resuspended in cell resuspension buffer at a concentration of 1,000 nuclei per μL for library preparation. The 10 × Genomics system was employed for library preparation and libraries were sequenced on NovaSeq System (Illumina) by Kidio Biotechnology Co., Ltd. (Guangzhou, China). After filtering raw reads, clean reads were aligned to the chicken reference genome. An expression matrix was generated with three files including genes, barcodes, and raw UMI count by Cell Ranger software and then a Seurat object was created using the Seurat R package. Cells were kept for each sample if the percentage of

mitochondrial genes was less than 10%, the number of identified genes was more than 200, and the UMI count was less than 8800. Following removing low-quality cells, Harmony was employed for data integration and batch effect correction. Dimension reduction, clustering, and visualization were conducted using default parameters. Genes were identified as marker genes if they were detected in at least 25% of the cells within a cluster with a P value < 0.01 and an absolute log2 fold change > 0.36. Each cluster was annotated based on the expression of established marker genes.

Preparation of lymphocytes and flow cytometry

The preparation of single-cell suspensions from the terminal ileum and the intracellular staining of *IL-17A* were performed as described in previous studies [7, 8]. Primary antibodies against mouse anti-chicken CD45-APC (Southernbiotech, 8270-11), mouse anti-chicken CD3-Alexa Fluor 700 (Southernbiotech, 8200-27), mouse anti-chicken CD4-PE/CY7 (Southernbiotech, 8210-17), human anti-chicken CD25-Alexa Fluor 647 (Bio-Rad, HCA173A647), and rat anti-mouse IL17A-PE (Biolegend, 506904) were used in the flow cytometry assays. Cells were acquired with a spectral cell analyzer (SONY ID7000), and analysis was performed using FlowJo software.

Microscopic observation

The terminal ileum samples fixed in 4% paraformaldehyde were embedded in paraffin and stained with hematoxylin and eosin. Five random visual fields of per section were captured under the microscope at 40 × magnification, and the villus height and crypt depth were measured in each visual field. Specifically, the SFB were observed under the microscope at 400 × magnification. The Gram staining was performed using the

Gram Stain Kit (Solaibao, China) according to the manufacturer's instructions, and the SFB were observed under the microscope at 1000 × magnification. The 0.5 cm sections of the terminal ileum were dissected, fixed in a fixative solution containing 2.5% glutaraldehyde, processed according to standard procedures, critical point dried, and treated with gold spraying for scanning electron microscopic observation.

Metagenomic sequencing and chicken SFB genome assembly

The metagenomic DNA from the terminal ileum contents of 7-day-old SynCom^{Bac10} chickens was extracted using a QIAGEN DNeasy PowerSoil Pro Kit (Qiagen Ltd., Dusseldorf, North Rhine-Westphalia, Germany) and sequenced using the DNBSEQ-T7 System (Ling En Biotechnology Co., Ltd., Shanghai, China). A total of 102,995,204 shotgun metagenomic sequencing read pairs were obtained. Following assembly, 124,673 contigs were generated and used for genome binning. After filtering the low-quality reads using Trimmomatic, the clean reads were assembled into genomes by Megahit. The MetaWRAP integrating CONCOCT, MaxBin2, and MetaBAT2 was used for binning, and the dRep tool was utilized to remove duplicate bins. Taxonomic annotation was conducted with GTDB-Tk, and relative abundance estimations were calculated via CoverM. The completeness and contamination of the genomes were assessed using Checkm2.

The SFB genomes of different host origins were downloaded from the NCBI or the European Nucleotide Archive (ENA). The SNPs were identified by the kSNP3 software, followed by the construction of a phylogenetic tree employing the Maximum Likelihood (ML) method on the iTOL platform (https://itol.embl.de/). The ANI and AAI among all SFB genomes were calculated using pyani and EzAAI,

respectively. Genomes with an ANI > 95% were considered to belong to the same species, and those with an AAI > 60% were deemed to be of the same genus [9]. Genome-scale metabolic capabilities were predicted using gapseq [10], and iNAP 2.0 was employed to identify potentially transferable metabolites from ten SynCom^{Bac10} members to SFB [11]. Proksee (https://proksee.ca) was applied to draw a genome map.

References

1. Weiss AS, Burrichter AG, Durai Raj AC, von Strempel A, Meng C, Kleigrewe K *et al*. *In vitro* interaction network of a synthetic gut bacterial community. *ISME J.* 2022;**16**:1095-109. https://doi.org/10.1038/s41396-021-01153-z

2. Redweik GA, Kogut MH, Arsenault RJ, Mellata M. Oral treatment with ileal spores triggers immunometabolic shifts in chicken gut. *Front Vet Sci*. 2020;**7**:629. https://doi.org/10.3389/fvets.2020.00629

3. Meinen-Jochum J, Ott LC, Mellata M. Segmented filamentous bacteria-based treatment to elicit protection against *Enterobacteriaceae* in Layer chickens. *Front Microbiol*. 2023;**14**:1231837. https://doi.org/10.3389/fmicb.2023.1231837

4. Hitch TC, Riedel T, Oren A, Overmann J, Lawley TD, Clavel T. Automated analysis of genomic sequences facilitates high-throughput and comprehensive description of bacteria. *ISME Commun*. 2021;**1**:16. https://doi.org/10.1038/s43705-021-00017-z

5. Feng Y, Zhang M, Liu Y, Yang X, Wei F, Jin X *et al.* Quantitative microbiome profiling reveals the developmental trajectory of the chicken gut microbiota and its connection to host metabolism. *iMeta*. 2023;**2**:e105. https://doi.org/10.1002/imt2.105

6. Gao P, Ma C, Sun Z, Wang L, Huang S, Su X *et al.* Feed-additive probiotics accelerate yet antibiotics delay intestinal microbiota maturation in broiler chicken. *Microbiome*. 2017;**5**:91. https://doi.org/10.1186/s40168-017-0315-1

7. Song B, Li P, Yan S, Liu Y, Gao M, Lv H *et al.* Effects of dietary astragalus polysaccharide supplementation on the Th17/Treg balance and the gut microbiota of broiler chickens challenged with necrotic enteritis. *Front Immunol*. 2022;**13**:781934. https://doi.org/10.3389/fimmu.2022.781934

8. Geva-Zatorsky N, Sefik E, Kua L, Pasman L, Tan TG, Ortiz-Lopez A *et al.* Mining the human gut microbiota for immunomodulatory organisms. *Cell*. 2017;**168**:928-43. https://doi.org/10.1016/j.cell.2017.01.022

9. Glendinning L, Stewart RD, Pallen MJ, Watson KA, Watson M. Assembly of hundreds of novel bacterial genomes from the chicken caecum. *Genome Biol*. 2020;**21**:34. https://doi.org/10.1186/s13059-020-1947-1

10. Zimmermann J, Kaleta C, Waschina S. Gapseq: informed prediction of bacterial metabolic pathways and reconstruction of accurate metabolic models. *Genome Biol*. 2021;**22**:81. https://doi.org/10.1186/s13059-021-02295-1

11. Peng X, Feng K, Yang X, He Q, Zhao B, Li T *et al.* iNAP 2.0: Harnessing metabolic complementarity in microbial network analysis. *iMeta*. 2024;**3**:e235. https://doi.org/10.1002/imt2.235

Supplementary Figures

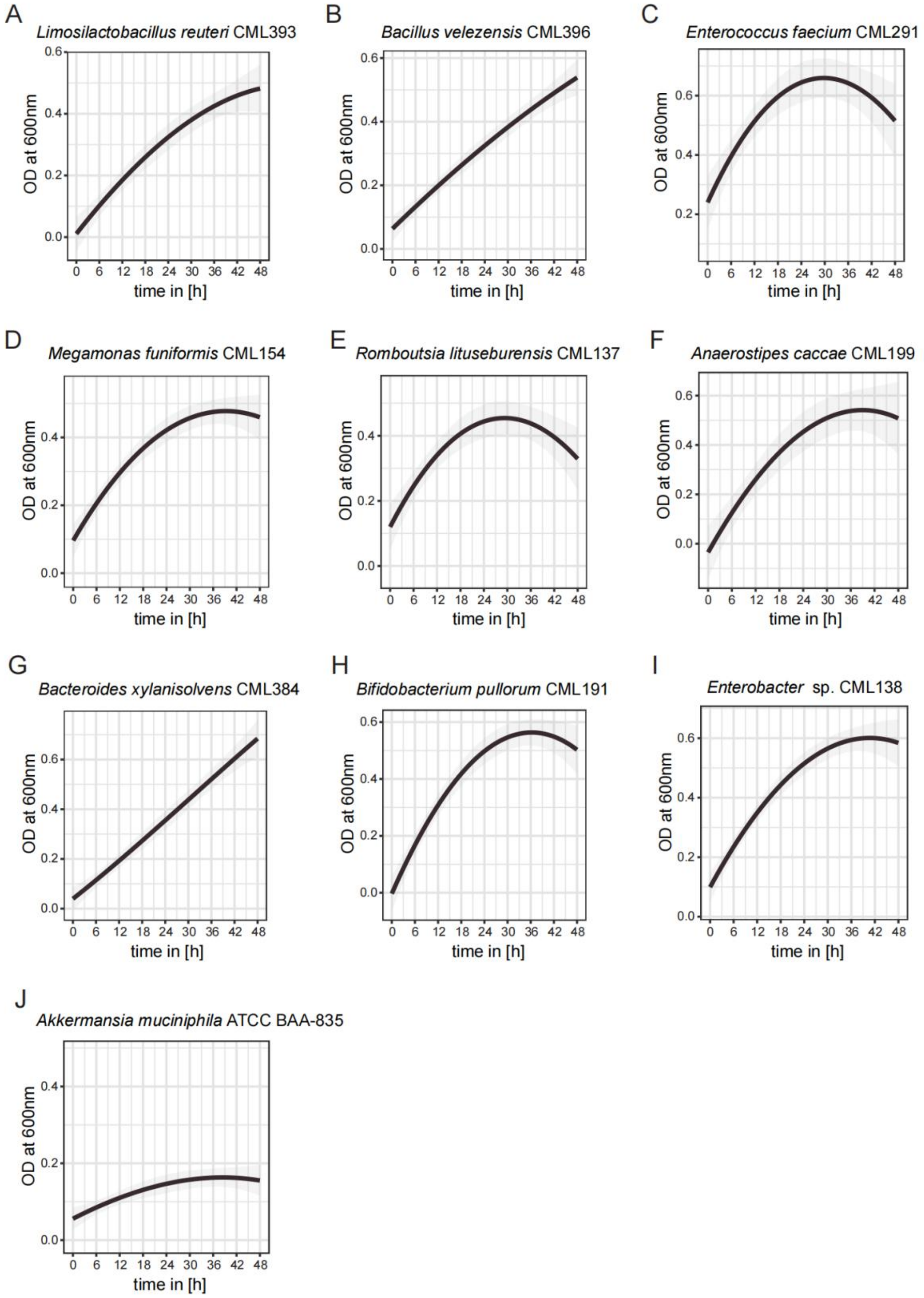

Fig. S1 Growth characteristic of individual strain in monoculture.

Growth of *Limosilactobacillus reuteri* CML393 **(A)**, *Bacillus velezensis* CML396 **(B)**, *Enterococcus faecium* CML291 **(C)**, *Megamonas funiformis* CML154 **(D)**, *Romboutsia lituseburensis* CML137 **(E)**, *Anaerostipes caccae* CML199 **(F)**,

Bacteroides xylanisolvens CML384 **(G)**, *Bifidobacterium pullorum* CML191 **(H)**, *Enterobacter* sp. CML138 **(I)**, and *Akkermansia muciniphila* ATCC BAA-835 **(J)** in GAM medium was monitored at OD_{600} nm within 48 hours, mean (black line) and SD (gray) of three independent experiments is shown.

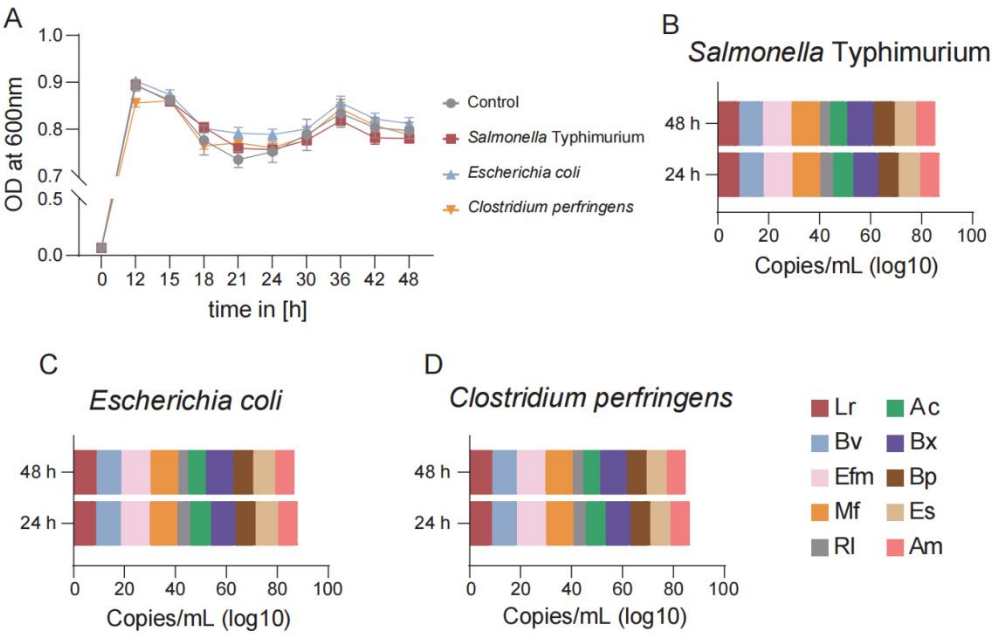

Fig. S2 Growth characteristics of SynCom$^{\text{Bac10}}$ strains under pathogens challenging.

(A) Growth of SynCom$^{\text{Bac10}}$ in GAM medium was monitored at OD_{600} nm within 48 hours under three pathogens challenging. SynCom$^{\text{Bac10}}$ composition was analyzed after 48 hours by comparing the absolute abundances of ten strains in GAM medium using RT-qPCR under *Salmonella* Typhimurium **(B)**, *Escherichia coli* **(C)**, and *Clostridium perfringens* **(D)** challenging.

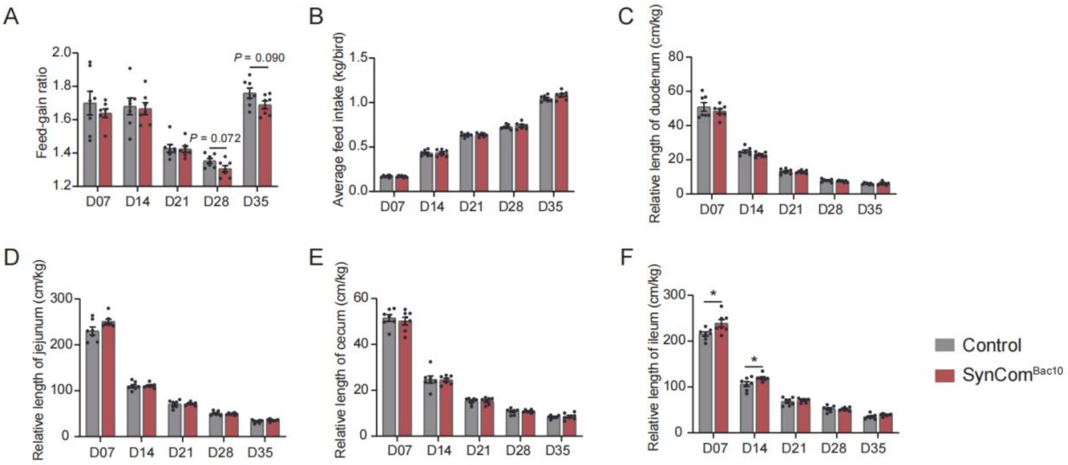

Fig. S3 Effects of SynCom^{Bac10} on chicken growth performance and gut health.

(A) Feed-gain ratio of chickens from day 7 to day 35. **(B)** Average feed intake of chickens from day 7 to day 35. Relative length of duodenum **(C)**, jejunum **(D)**, cecum **(E)**, and ileum **(F)** from day 7 to day 35. * P value < 0.05, ** P value < 0.01, and *** P value < 0.001.

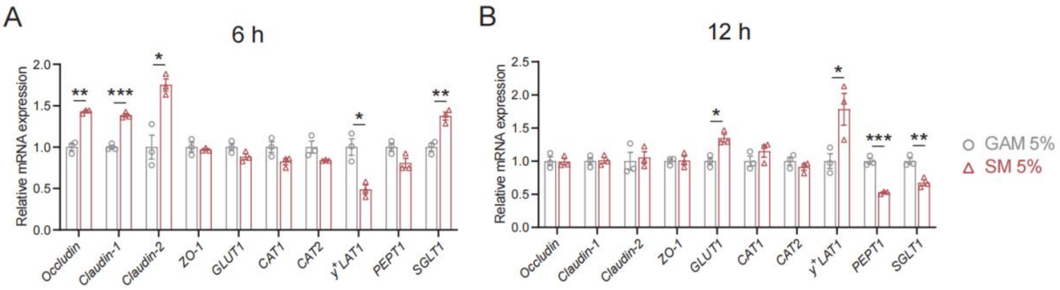

Fig. S4 Effects of SynCom^{Bac10} on primary chicken embryo IECs.

The relative mRNA expression of intestinal barrier proteins and nutrient transporters in IECs cultures containing 5% sterile fermentation supernatant of SynCom^{Bac10} at 6 h **(A)** and 12 h **(B)**. * *P* value < 0.05, ** *P* value < 0.01, and *** *P* value < 0.001.

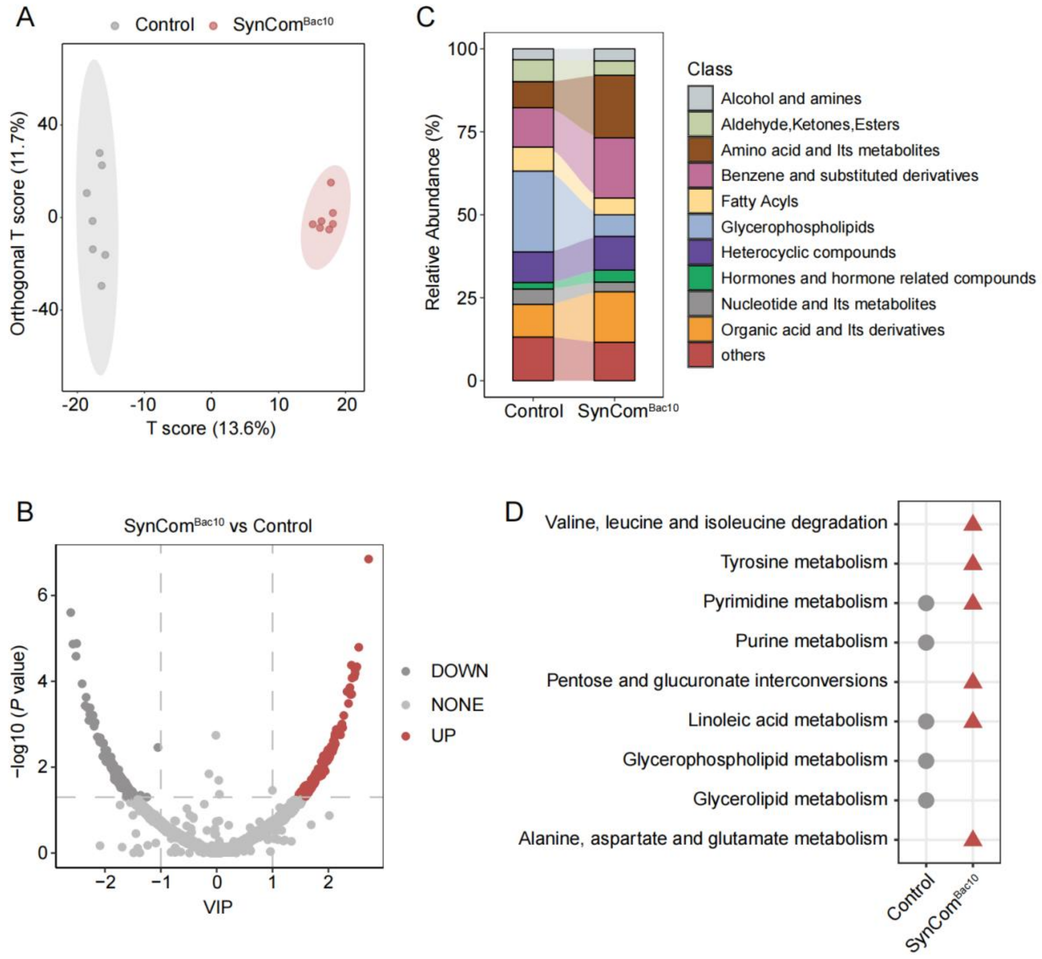

Fig. S5 Effects of SynCom^{Bac10} on serum metabolites.

(A) OPLS-DA of serum metabolites between the control and SynCom^{Bac10} chickens. **(B)** Volcano map of SCMs with VIP > 1 and *P* value < 0.05 between the control and SynCom^{Bac10} chickens. **(C)** The HMDB classification of SCMs. **(D)** KEGG enrichment analysis was implemented using the SCMs between the control and SynCom^{Bac10} chickens (*P* value < 0.05).

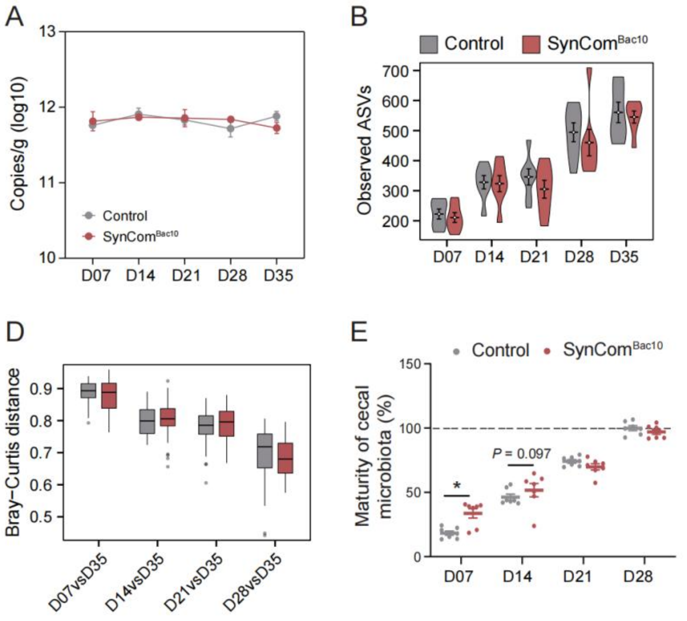

Fig. S6 SynCom^{Bac10} accelerates cecal microbiota maturation.

(A) Dynamics of bacteria in the cecum of the control and SynCom^{Bac10} chickens based on microbial absolute abundance. **(B)** Observed ASVs of cecal microbiota between the control and SynCom^{Bac10} chickens during 35 days. **(C)** Pairwise Bray-Curtis distances of cecal bacteria from adjacent sampling time points. **(D)** Bray-Curtis distances between the cecal bacteria at D35 and the other four sampling times. **(E)** Maturity of cecal microbiota between the control and SynCom^{Bac10} groups. * P value < 0.05, ** P value < 0.01, and *** P value < 0.001.

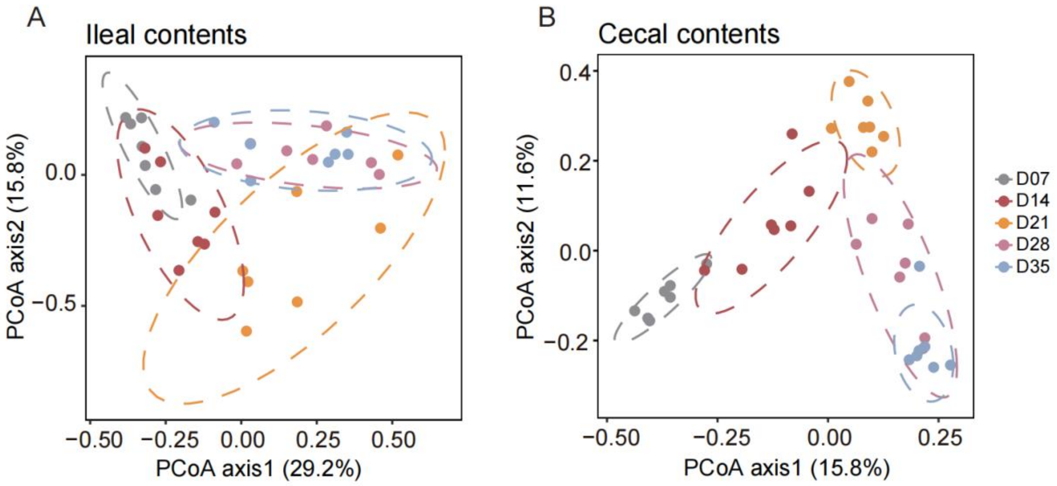

Fig. S7 The β-diversity of ileal and cecal microbiota under normal growth conditions.

(A) PCoA plot based on Bray-Curtis distance of ileal microbiota composition. **(B)** PCoA plot based on Bray-Curtis distance of cecal microbiota composition.

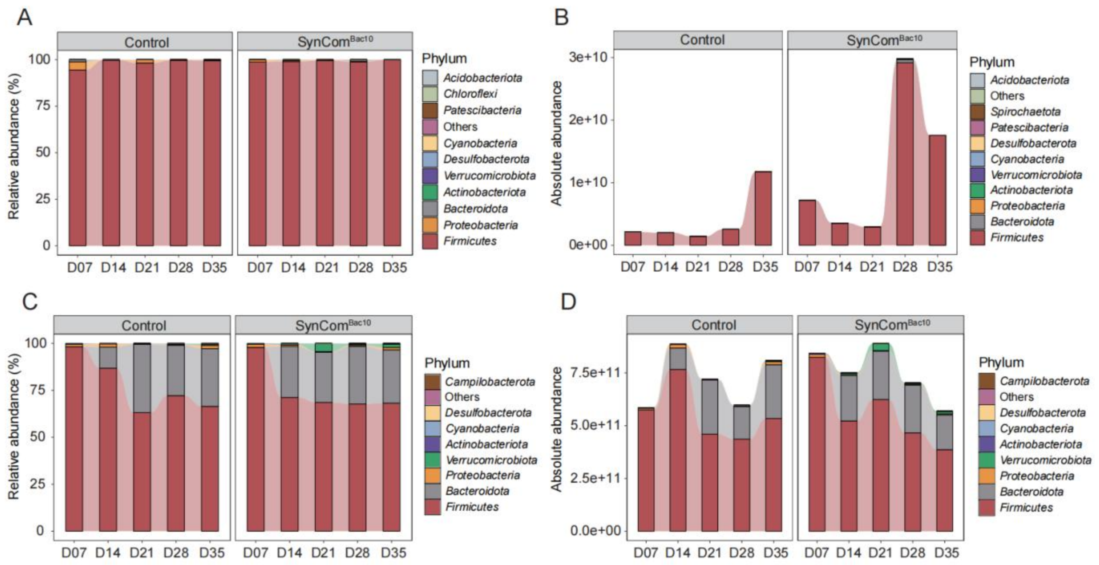

Fig. S8 Abundance dynamics of gut microbiota at the phylum level.

Relative abundance **(A)** and absolute abundance **(B)** of ileal microbiota in the control and SynCom^{Bac10} chickens at the phylum level. Relative abundance **(C)** and absolute abundance **(D)** of cecal microbiota in the control and SynCom^{Bac10} chickens at the phylum level.

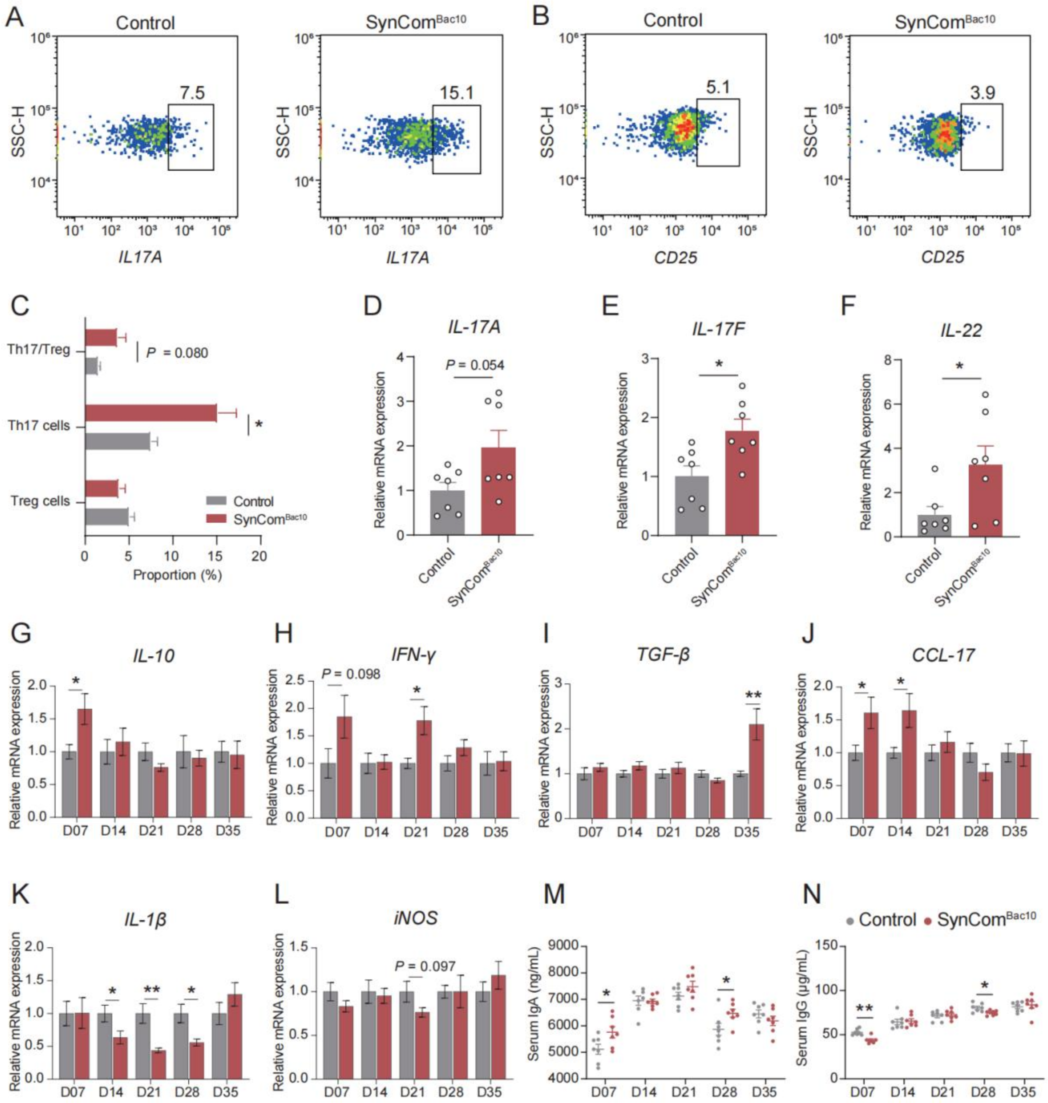

Fig. S9 The immune responses of chickens with SynCom^{Bac10} exposure.

Th17 **(A)** and Treg cells **(B)** differentiation in ileal lamina propria in the control and SynCom^{Bac10} chickens at D07. **(C)** The proportions of Th17, Treg cells, and their ratio in ileal lamina propria detected by flow cytometry. The mRNA levels of *IL-17A* **(D)**, *IL-17F* **(E)**, and *IL-22* **(F)** in the ileum at D07. The mRNA levels of *IL-10* **(G)**, *IFN-γ* **(H)**, *TGF-β* **(I)**, *CCL-17* **(J)**, *IL-1β* **(K)**, and *iNOS* **(L)** in the ileum from D07 to D35. **(M)** Serum IgA concentration. **(N)** Serum IgG concentration. * *P* value < 0.05, ** *P* value < 0.01, and *** *P* value < 0.001.

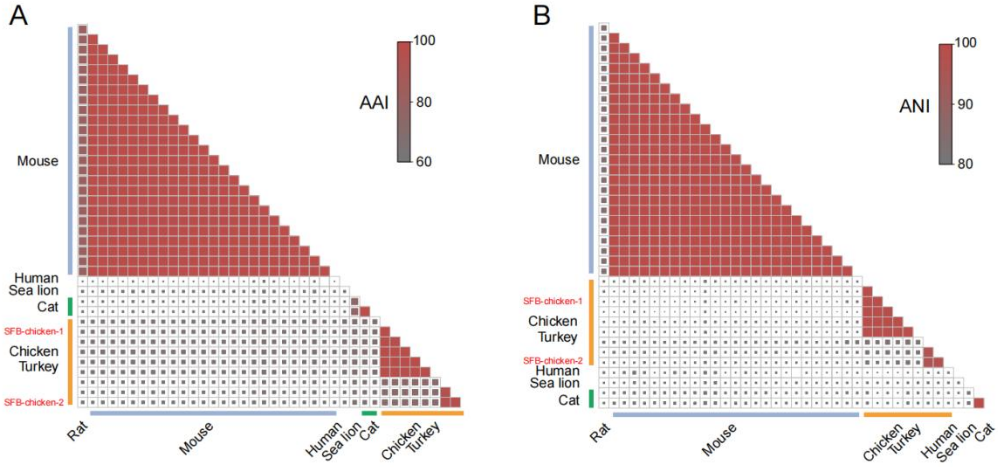

Fig. S10 The AAI and ANI analysis of SFB genomes from different host origins.

(A) Average amino acid identity (AAI) between SFB genome pairs. **(B)** Average nucleotide identity (ANI) between SFB genome pairs.

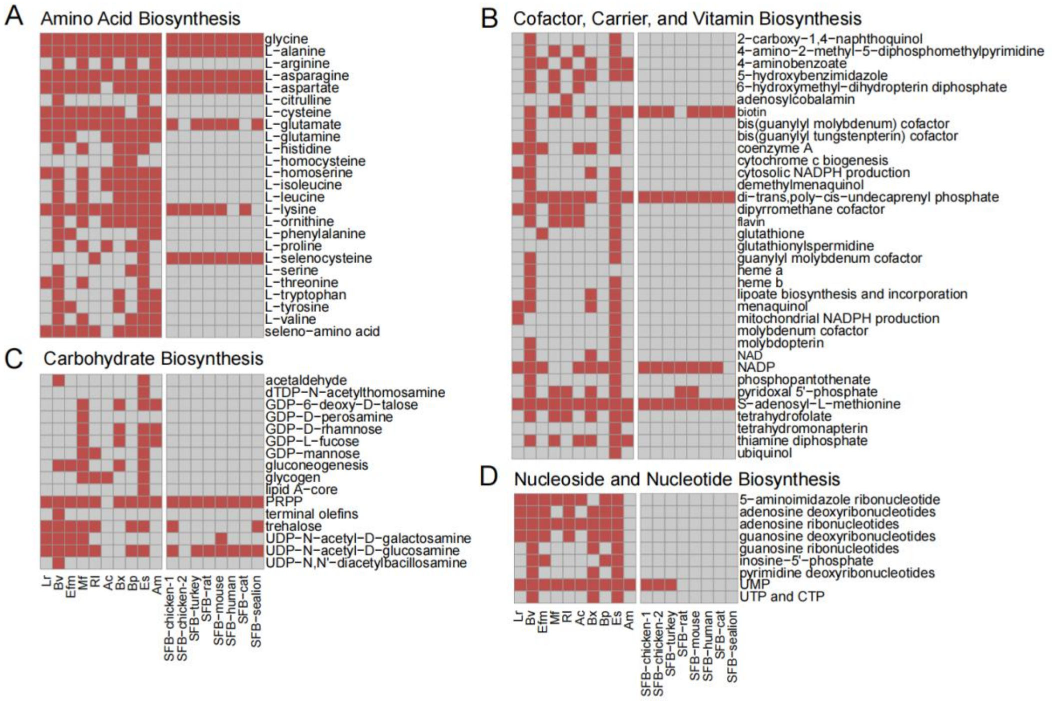

Fig. S11 Metabolic profiles of SFB and SynCom^{Bac10} members.

Amino acid biosynthesis **(A)**, cofactor, carrier, and vitamin biosynthesis **(B),** carbohydrate biosynthesis **(C)**, and nucleoside and nucleotide biosynthesis **(D)** pathways in eight SFB strains (right panels) and ten SynCom^{Bac10} members (left panels) were predicted using gapseq tool. Red rectangles mean the presence of the pathway, and gray rectangles mean the absence of the pathway.
